# Associations between touchscreen exposure and hot and cool inhibitory control in 10-month-old infants

**DOI:** 10.1016/j.infbeh.2021.101649

**Published:** 2021-11

**Authors:** Katie Y.K. Lui, Alexandra Hendry, Abigail Fiske, Henrik Dvergsdal, Karla Holmboe

**Affiliations:** a Department of Psychology, University of Bath, Bath, United Kingdom; b Department of Experimental Psychology, University of Oxford, Oxford, United Kingdom; c Nord University Business School, Department of Entrepreneurship, Innovation and Organisation, Bodø, Norway; d School of Psychological Science, University of Bristol, Bristol, United Kingdom

**Keywords:** Screen media, Touchscreen, Infant cognition, Inhibitory control, Executive functions

## Abstract

Touchscreen use amongst young children has proliferated in recent years, yet little is known about the association between daily touchscreen exposure and inhibitory control in the first year of life. Previous research has found a negative association between the amount of television viewing and inhibitory control in early childhood, but it is unclear whether negative associations with screen use extend to touchscreens. The current study presents an exploratory analysis of the cross-sectional associations between inhibitory control and the amount of touchscreen use amongst 10-month-olds (*n* = 128–156). Touchscreen exposure was assessed via parent-report. In order to include a range of “hot” and “cool” aspects of inhibitory control, these skills were assessed using lab-based response inhibition and prohibition tasks as well as parent-reported observations of infants’ inhibitory control abilities and broader regulatory behaviors. A “Cognitive Executive Function (EEFQ-CEF)” score (encompassing Inhibitory Control, Flexibility, and Working Memory items) was included as a secondary broader executive function construct to examine whether effects showed specificity to inhibitory control rather than executive functions more generally. Correlation analyses indicated no association between touchscreen exposure and the four indices of IC. However, a positive association was found for the amount of touchscreen exposure and EEFQ-CEF once accounting for sociodemographic variables. The implications of these findings and future directions are discussed.

## Highlights

• No association between touchscreen exposure and inhibitory control at 10-months.
• Touchscreen exposure positively associated with Cognitive Executive Function score.
• Positive association found only after accounting for sociodemographic variables.
• Touchscreen exposure during infancy may be less detrimental than previously believed.

## 1 Introduction

For many young children, the use of screen-based media has become a daily activity. Children under the age of two in the United States spend an average of 2.62 h watching television and 0.37 h using smartphones per day (Chen & Adler, 2019). In a recent UK study of 715 children aged 6–36 months, 75 % were reported by parents to use a touchscreen device daily, and 51 % of the sample first used touchscreens when they were aged 6–11 months (Bedford et al., 2016). The prevalence of screen-based media in the lives of young children may be cause for concern as guidelines proposed by the American Academy of Pediatrics (AAP, 2016) state that children under 18 months should avoid all forms of screen media except video chatting. The World Health Organization (WHO, 2019) has even stricter recommendations: children under 2 should not be allowed any screen time. This may, in part, explain why children from the US were reported to have lower screen time and thus 30 % less likely to be considered “high media users” compared to children from the UK (Ribner & McHarg, 2021). Therefore, these guidelines hold meaningful implications as they may dictate the amount of screen time parents from different countries allow for their children.

However, the evidence supporting these recommendations remains scant and is primarily based on research involving traditionally established screen-based platforms (i.e., television; Christakis, 2014). Although there is accumulating evidence highlighting the detrimental effects of screen-based media on cognitive development (for review, see Kostyrka-Allchorne et al., 2017), it is unclear whether the purported harmful effects of televisions extend to mobile devices (i.e., smartphones and tablets). Given that a recent survey by Common Sense Media (2017) reported a rise in touchscreen usage and a slight decline in television viewing (from an average of 44 min per day in 2013 to 29 min per day in 2017) amongst children aged 0–2, it is important to determine whether the AAP and WHO guidelines should be modified to provide more nuanced advice on the benefits and risks of screen use in infancy.

While touchscreens may be similar to televisions in their ability to stream content (Kabali et al., 2015), touchscreens have the potential to offer a multimodal stimulation that televisions do not (Cristia & Seidl, 2015). Mobile devices have also enabled exposure to interactive screen-based media to occur at a much earlier age, especially as these devices can be easily navigated via simple swiping motions (Ahearne et al., 2016); thus, increasing their accessibility for young infants with limited fine motor control (Cristia & Seidl, 2015). Despite the earlier age of onset and the dramatic increase in touchscreen use (Chassiakos et al., 2016), the majority of studies have focused on the use of screen-based media in toddlers (2–3 years), preschoolers (3–5 years), and school-aged children (6–8 years) (e.g., Linebarger et al., 2014; McHarg et al., 2020b; McNeill et al., 2019). The potential impact of early touchscreen exposure on cognitive development may vary in important ways for infants under 1 year of age as infants could be engaging with screens quite differently (Cheung, 2016). For example, there is evidence that children aged 6- to 24-months use touchscreens most commonly for video chatting (McClure et al., 2015), whereas 32- to 40-month-olds mainly use them for viewing photos, followed by watching videos, then playing with apps (Cristia & Seidl, 2015). Given that video chatting often occurs in the presence of an adult (76 %) (McClure et al., 2015), it is possible that parental co-viewing may buffer against the detrimental effects of screen exposure (Christakis, 2009).

Although screen use has been linked to many aspects of early childhood development (e.g., language, health, sleep; for reviews, see Duch et al., 2013; Hale & Guan, 2015), research in recent years has highlighted potential associations with young children’s emerging executive function skills; in particular, the ability to exert inhibitory control (Barr et al., 2010; McHarg et al., 2020a). Executive functions (EFs) refer to core cognitive skills needed to control and guide behavior, commonly including inhibitory control, working memory, and cognitive flexibility (Diamond, 2013; Lehto et al., 2003). Inhibitory control (IC) specifically refers to the ability to suppress a thought or response to achieve an optimal outcome (Diamond, 2013), and can be characterized as either “hot” or “cool”. Hot IC serves in more emotionally salient contexts (Zelazo & Carlson, 2012), such as the ability to resist temptation (e.g., delay of gratification or following a prohibition given by an adult). Meanwhile, cool IC refers to cognitive skills typically assessed by more abstract tasks (Brock et al., 2009), such as the ability to overcome a prepotent motor response to make an alternative response (i.e., response inhibition). Hot IC has previously been linked to regulatory behaviors, such as the ability to self-soothe and modulate emotional arousal (Kochanska et al., 1996, 2000; Rothbart et al., 2006). As such, self-regulation could be considered to have a particularly strong “hot” IC component (Di Norcia et al., 2015; Kim et al., 2013).

Understanding the potential effects of screen exposure on IC development is of great importance as IC abilities have been linked to later academic performance and social skills (for reviews, see Allan et al., 2014; Schoemaker et al., 2013). Of note, infants may not comprehend child-directed content from televisions until 2 years of age (Anderson & Subrahmanyam, 2017; Hipp et al., 2017), and lack the ability to process screens for more than 3–5 s (for summary, see Kirkorian et al., 2017). Owing to infants’ and toddlers’ limited comprehension skills, early screen exposure may therefore be considered adult-directed content (Anderson & Subrahmanyam, 2017). This is important as higher exposure to adult-directed content at 12- to 18-months has been linked to poorer IC at age 4 years (Barr et al., 2010), suggesting that the adverse impact of screen exposure could potentially affect infant’s EF development more strongly than in older children. Indeed, a study that examined the effects of screen use during toddlerhood found that while higher screen use at age 2 predicted lower EF a year later, no association was found between concurrent screen time and EF at age 3 (McHarg et al., 2020b), implying that early screen exposure may be more detrimental to EF development compared to later exposure.

However, the potentially adverse effects of screens may be specific to, or stronger for, certain EF domains, especially when screen use begins during infancy. McHarg et al. (2020a) found that regular screen exposure (including TVs, tablets, and computers) at 4-months predicted poorer IC at 14-months, but not working memory and cognitive flexibility. Interestingly, although there was clear evidence of a group-level effect, there was no linear association between the *amount* of screen exposure at 4-months and IC at 14-months. In other words, infants who had not been exposed to screens at all exhibited better IC than infants who, on a typical day, had some level of screen exposure, suggesting that even a small amount of screen exposure is detrimental to IC development. Yet, it is unclear whether these findings extend to cool IC measures, as IC in the study by McHarg et al. (2020a) was measured using a single-trial prohibition task (i.e., hot IC). Given that the first IC skills emerge as early as 6 months of age (Holmboe et al., 2018), it is important to examine the association between touchscreen usage and developing IC skills during the first year of life using a wider range of measures covering both hot and cool aspects of IC.

So far, the association between touchscreen use and infant IC remains relatively understudied, but some insights into potential effects may be gained by considering research on the effects of screen exposure in slightly older children. Experimental studies have revealed immediate adverse effects of television viewing on preschoolers’ IC. Four-year-olds who watched a cartoon involving fantastical events were less likely to delay gratification (a type of hot IC) compared to those who watched realistic events (Lillard & Peterson, 2011; Lillard et al., 2015). However, these disruptive effects were mitigated when children interacted with these events in the form of a touchscreen game (Li et al., 2018). The interactive aspect of touchscreens may, therefore, potentially counteract the negative effects of screen-based media on IC.

Similar results have been found in toddlers: 2- to 3-year-olds were less likely to delay gratification (a hot IC task) after viewing a cartoon compared to those playing with an educational app. Interestingly, the adverse effects of viewing cartoons (compared with the educational app) were not found when assessed by a cool IC task (Huber et al., 2018). While the authors speculated that the null findings on the cool IC measure were due to the task familiarity gained during the baseline practice, it is possible that the benefits of screen interactivity may affect various types of IC and age groups differentially. Indeed, no association was found between response inhibition skills and amount of screen use (including TVs, computers, and touchscreen devices) in preschool children (Jusienė et al., 2020). More research investigating the potential impact of touchscreen interfaces on both hot and cool IC development is needed to determine whether touchscreens may be less detrimental for IC compared to passive screen exposure.

With regards to associations between screen use and broader regulatory skills, some research indicates that frequent and early exposure to touchscreens may disrupt self-regulation in young children. Coyne et al. (2021) found that toddlers (age 2–3 years) who are often given a media device to manage their difficult emotions tend to show poorer self-regulation. The authors speculated that the over-reliance on media may subsequently hinder their learning of positive emotion-regulation strategies. Similarly, Lawrence et al. (2020) found that 32- to 47-month-olds who use mobile devices more regularly or started using screen-based media of any kind at an earlier age exhibited lower self-regulation when assessed by an 11-task behavioral battery that included hot IC tasks (e.g., Gift Delay); although not when assessed by parental report. These findings indicate that both the amount and the age of initial touchscreen exposure may be linked to self-regulation. More consideration should therefore be given to these two factors when assessing children’s daily touchscreen exposure and its subsequent effects on hot IC. Furthermore, both laboratory and parent-report measures should be included when evaluating associations with IC-related constructs, as results may differ across these assessment contexts.

While extant studies tend to emphasize the harmful effects of screen exposure, a growing body of literature has highlighted the potential benefits of early touchscreen use on child development. A recent study revealed that children who use touchscreens for longer durations at 12-months not only performed just as well as low touchscreen users on an anti-saccade task (which could be considered an IC task), but also made more corrective saccades when assessed at 18- and 42-months (Portugal et al., 2021). Children who had greater touchscreen exposure at 12-months were also faster in another attentional task (gap-overlap task; Portugal et al., 2021), thereby suggesting that touchscreens may facilitate faster attention allocation to salient stimuli. In addition, active scrolling on touchscreens at an earlier age was positively associated with fine motor achievement, an association not found for watching videos (Bedford et al., 2016). These findings again suggest that the age and type of touchscreen engagement are important factors to consider when assessing its potential role in childhood development. Apart from the purported benefits of interactive touchscreen engagement, the visual and social engagement involved in some uses of touchscreens, such as video chatting, may also promote positive child development. Indeed, Roseberry et al. (2013) found that the social contingency offered through video chatting could facilitate language learning in young children. Although the AAP guidelines were revised in 2016 to remove video calls from their recommended media restrictions for young children (AAP, 1999, 2016), these studies contradict the strong narrative in the popular press regarding the negative impacts of early screen use (e.g., Davis, 2018; Ortiz, 2018; Weale, 2016).

In summary, our knowledge regarding touchscreen use in infancy is limited. We know little about how the amount of touchscreen exposure could affect the hot and cool aspects of IC in infants in their first year of life. We also need confirmation of previous infant studies linking screen use and early IC development (e.g., McHarg et al., 2020a) using broader measures than a single prohibition trial. Finally, it remains an open question whether touchscreen exposure in infancy is as detrimental (or more) for early IC development as some forms of television (e.g., Barr et al., 2010; Nathanson et al., 2014), or whether the interactivity and screen devices’ potential to facilitate social interaction prevent some of these negative effects or may even be beneficial to early IC development.

To address these questions, the present study aimed to examine the potential association between the amount of daily touchscreen exposure and IC skills in 10-month-old infants. Touchscreen use was captured as the amount of current exposure and age of initial exposure, assessed via a brief parental questionnaire addressing various types of engagement, including passive, active, and social engagement (e.g., watching videos, playing with simple apps, and video chatting). Touchscreen usage in this paper is therefore conceptualized as a broad construct involving aspects of passive, active, and social activity on a smartphone, tablet, or any other touchscreen interface. Note that viewing of traditional non-touch devices, such as TVs, was not considered. IC was assessed by two experimental tasks and two subscales of a parent-report questionnaire. The use of four indices enabled us to explore several aspects of IC: parent-reported regulation (hot IC), compliance with prohibition (hot IC), parent-reported IC (mixed hot and cool IC), and response inhibition (cool IC). By using both behavioral measures and parental report, we were able to consider two potentially quite different aspects of IC: the ability to exert IC in a controlled but unfamiliar setting (behavioral tasks), and the ability to exert IC in day-to-day life (parental report). A composite Cognitive Executive Function (EEFQ-CEF) score encompassing Inhibitory Control, Flexibility, and Working Memory items (Hendry & Holmboe, 2021) was also included as a secondary broader EF construct to examine whether effects showed specificity to IC rather than involving EFs more generally, as might be expected based on the literature.

## 2 Method

### 2.1 Participants

Participants were 10-month-old infants (*N* = 163; 85 boys) who were taking part in a longitudinal study on early executive function development in the United Kingdom. The 10-month assessment was the first in the longitudinal study. Demographic characteristics of the sample are reported in Table 1. Data collection took place between April 2019 and May 2020, where testing sessions happened within one week before and two weeks after infants reaching their 10-month birthday. Parents gave informed consent, and the study received ethical approval from the Medical Sciences Inter-Divisional Research Ethics Committee at the University of Oxford (Ref. No. R57972/RE001). Participants were recruited from the local hospital, email from the Oxford University BabyLab volunteer database, social media posts, or word of mouth. Participants were only included if they met at least one of the following criteria: (a) born at 36 weeks’ gestation or later or (b) weighing at least 5.5 lbs (2.5 kg) at birth. Three participants were excluded from analysis due to potentially serious health issues (e.g., brain abnormalities, oxygen deprivation at birth). Parents received a £20 digital Amazon voucher and infants received a small gift as a token of appreciation for their time and effort.

**Table 1:** Demographic Characteristics of the Sample.

Characteristic	*N*	Mean	*SD*	Min	Max
Infant’s age (in days)	163	305	6.63	293	326
Mother’s age (in years)	161	34.07	4.48	19	47
Father’s age (in years)	158	35.73	5.41	22	53
Maternal education (in years)	155	17.90	3.15	10	28
Paternal education (in years)	145	17.28	3.26	9	30
*Infant Ethnicity*					
White	136	(83.44 %)			
Mixed White-Asian	7	(4.29 %)			
Mixed White-African/Caribbean	6	(3.68 %)			
Other Mixed background	3	(1.84 %)			
Asian	5	(3.07 %)			
Other Ethnic background	4	(2.45 %)			
Prefer not to answer	2*	(1.23 %)			

*Note.* Numbers in brackets indicate the frequency of a characteristic (%) in the current sample.

* One missing response was collapsed into the same group as “prefer not to answer.”

Performance in the current sample on the Early Childhood Inhibitory Touchscreen Task (ECITT) has been previously reported in Fiske et al. (2021) and Early Executive Function Questionnaire (EEFQ) data from the current sample is included in Hendry and Holmboe (2021). However, neither dataset has been analyzed with regards to touchscreen use.

### 2.2 Measures

#### 2.2.1 Touchscreen Use Questionnaire (TUQ)

##### 2.2.1.1 Procedure

The Touchscreen Use Questionnaire (TUQ) is a novel 12-item scale developed to assess the amount and age of initial touchscreen exposure in infants and toddlers (see SM 1). Parents completed this questionnaire while infants were completing a focused attention task during their lab visit. A total of 13 parents did not complete the TUQ, resulting in a sample of *n* = 150 with available TUQ scale scores. Note that both the testers and coders involved in the study were blind to parent-reported touchscreen use.

##### 2.2.1.2 Amount of Touchscreen Exposure Scale

Parents first rated the frequency with which their child engaged with a smartphone, tablet, or other touchscreen devices for performing the following actions: (a) watch videos or look at photos without touching the screen (i.e., with an adult controlling the screen), (b) scroll/swipe through photos or videos, (c) have video calls with loved ones, (d) play simple games (e.g., tapping or swiping a cartoon), and (e) do drawings/scribbles; on a 7-point Likert scale (1 = *Never*, 2 = *Once per month or less,* 3 = *2–4 times per month*, 4 = *5–8 times per month*, 5 = *More than twice per wee*k, 6 = *2–4 times per week*, 7 = *Most days*). Parents then reported the total duration that their child spent only looking at touchscreens and only interacting with touchscreens in the past week as two separate questions on the same 7-point scale (1 = *Less than 5 min*, 2 = *5–20 min,* 3 = *20–60 min*, 4 = *1–2 h*, 5 = *2–4 h*, 6 = *4–6 h*, 7 = *7 or more hours*). Scale scores were calculated by computing the mean of the seven items, where higher scores indicated greater touchscreen exposure. This 7-item scale had satisfactory internal consistency (α = .69).

##### 2.2.1.3 Age of Initial Touchscreen Exposure Scale

Parents reported the age their child first performed the five touchscreen actions (a) – (e) mentioned above on a 4-point Likert scale (1 = *Before 6 months,* 2 = *6–9 months*, 3 = *9–12 months*, 4 = *Has not done this yet*; note that action (a) was worded as “watch videos on a phone or tablet” on this scale). The scale was originally administered as an 8-point Likert scale to account for the other age points in the longitudinal study (1 = *Before 6 months*, 2 = 6*–9 months,* 3 = *9–12 months*, 4 = *12–15 months*, 5 =*15–18 months*, 6 = *18–21 months*, 7 = *21–24 months*, 8 = *Has not done this yet*), but at the time of responding there were only four feasible options for the 10-month-old participants (items 1, 2, 3, and 8). Therefore, the scores were computed and analyzed as a 4-point scale. Item scorings were then reversed so that higher scores indicated earlier touchscreen engagement, and scale scores were calculated by computing the mean of the five items. This 5-item scale, however, demonstrated poor internal consistency (α = .51). Analysis of the individual items indicated that this was due to substantial variability in when infants were first exposed to the different types of content. For example, whereas 39 % of infants had been able to watch videos on a touchscreen device from less than 6 months of age, only 4 % had played simple games at such a young age (for details, see SM 2, Supplementary Table 1.2). As the Age of Initial Touchscreen Exposure scale did not form a coherent and reliable scale, it was dropped from the main analyses. However, to explore whether specific items account for variance in IC performance, hierarchical multiple regression models were performed (for details, see Section 3.4). Correlations among the individual items from both scales are also provided (for the correlation matrix of items on the TUQ, see SM 3, Supplementary Table 3.1).

##### 2.2.1.4 Additional item

One question asked parents to rate the level of enjoyment their child experienced when playing with touchscreen interfaces (e.g., playing games or just tapping/swiping through photos and videos) on a 5-point scale (1 = *Not at all,* 5 = *A great deal*). As this item does not measure infant touchscreen exposure, it was not included in the final scales (for full details, see SM 2, Supplementary Table 2).

#### 2.2.2 Early Childhood Inhibitory Touchscreen Task (ECITT)

##### 2.2.2.1 Design and stimuli

The Early Childhood Inhibitory Touchscreen Task (ECITT; for full details, see Holmboe et al., 2021) was used to assess response inhibition, a type of cool IC. In this task, participants have to overcome the tendency to touch a frequently rewarded location (prepotent trials) in order to make an alternative response on the rarer inhibitory trials. The stimuli were presented as two blue rectangle-shaped buttons, one on the right and one on the left side of the screen, against a dark grey background. In each trial, a smiley icon appeared on either the left or the right button. The smiley appeared in one location on 24 trials (prepotent trials, 75 % of the block) and in the other location on 8 trials (inhibitory trials, 25 % of the block), randomized with the constraint that the smiley only appeared in the inhibitory location a maximum of two trials in a row. The first 3 trials were always prepotent (to ensure that the initial build-up of a prepotency was established). The prepotent location (either left or right) was manually counterbalanced across participants. Note that the response locations in Holmboe et al. (2021) were presented vertically (blue buttons at the top and bottom of the screen), whereas here they were presented horizontally. This was done because piloting indicated that it was easier for infants to reach horizontally presented stimuli.

Furthermore, in contrast to Holmboe et al. (2021, Studies 1, 3 and 4), stimuli did not disappear if the infant pressed the incorrect (blank) button. Instead, infants had to press the correct button (with the smiley on it) for the trial sequence to continue. This was done to make pressing the incorrect button minimally rewarding (no effect of touching). A touch on the correct button (with the smiley on) activated a short animation (e.g., elephant taking a shower) and a simple sound effect or tune. Finally, if infants pressed the pre-determined inhibitory location on the first trial, the inhibitory side was switched so that this side now became the prepotent location for the rest of the task. This was done because piloting indicated that first responses often became highly prepotent, with infants likely to perseverate on the first location they touch on the screen. Previous work indicates that in older samples ECITT performance is associated with performance on other well-established IC measures (Hendry et al., 2021; Holmboe et al., 2021), and data from both the current and a similarly sized independent sample have indicated that the task works well in 10-month-olds (Fiske et al., 2021; Hendry et al., 2021). Acceptable 1-week test-retest reliability has also been established for this task in the previous (*r*(48) = .30, *p* = .018, [*CI* = .032, .514]; Hendry et al., 2021) and current sample (*r*(110) = .48, *p* < .001, [*CI* = .296, .638]; Fiske et al., 2021). Therefore, the ECITT may be considered a valid and reliable measure of response inhibition in this age group.

##### 2.2.2.2 Procedure

Infants sat on the lap of their caregiver while an experimenter administered the ECITT on an iPad. The experimenter demonstrated the first trial by pressing the smiley face (which appeared on a single, centrally placed, button during practice trials). In the second practice trial, the experimenter encouraged the child to “press the happy face.” After two practice trials (or more if the infant was reluctant to touch the screen), a block of 32 experimental trials began. The experimenter was required to take the tablet back as soon as a correct response was made, as infants would otherwise sometimes not remove their hand from the screen between trials or would bash the screen repeatedly, resulting in too many invalid trials. However, experimenters took care to make sure that the screen was back within reach of the infant by the time the next trial started.

##### 2.2.2.3 Data preparation and scoring

To determine infants’ IC performance, the percentage of correct prepotent and inhibitory trials for each participant was first calculated. An adjusted accuracy difference score (adjusted AccD) was then calculated by subtracting the percentage of inhibitory trials responded to correctly from the percentage of prepotent trials responded to correctly, divided by the percentage of correct prepotent trials [(prepotent accuracy – inhibitory accuracy)/prepotent accuracy]. Note that this measure slightly differs from that reported in Holmboe et al. (2021) (prepotent accuracy – inhibitory accuracy) to control for situations where infants scored low on both the prepotent and inhibitory trials (i.e., the adjusted score accounts for the infants’ performance on the prepotent trials). Furthermore, to overcome the problem of generating negative correlations that result from a difference score (i.e., higher adjusted AccD indicates *lower* IC), the adjusted AccD was reversed (1 – adjusted AccD) to generate an ‘inhibitory score’ variable. A higher inhibitory score (i.e., reversed adjusted AccD) indicates *higher IC*.

The accuracy and response time (in milliseconds) of each trial was recorded by the iPad and were coded offline to ensure that responses were recorded correctly by the software (in some instances, a light or swiping touch was not detected, and this was corrected during coding). Coders were also required to judge the validity of each trial based on a detailed coding protocol (for full protocol, see SM 4 and SM 5). Invalid trials were subsequently excluded from further analysis.

Double coding of 17 videos (534 trials) by two independent coders established excellent intercoder reliability (κ = .97 for trial accuracy; κ = .93 for trial validity). Based on the same 17 participants, a third coder also established excellent intercoder reliability (κ = .97 for accuracy; κ = .88 for validity) with the final agreed coding of the first two coders.

In accordance with Holmboe et al. (2021), infants were excluded if they were less than 60 % correct on the prepotent trials (*n* = 9) or did not complete at least 16 trials with a minimum of two valid inhibitory trials (*n* = 4). The requirement for infants to be at least 60 % correct on prepotent trials was necessary to ensure that a prepotency was built up during the task in the first place. Otherwise, performance on the inhibitory trials could not be interpreted as having an inhibitory demand. As mentioned in Section 2.2.2.1, if infants showed an immediate preference for the inhibitory side at the beginning of the task, the sides were switched. A subset of parents completed the parental questionnaires but did not attend the lab visit (*n* = 22), resulting in a final sample with ECITT data available of *n* = 128.

#### 2.2.3 Toy Prohibition (TP)

##### 2.2.3.1 Procedure

In the Toy Prohibition (TP) task (Friedman et al., 2011), the child was seated on their caregiver’s lap while the experimenter captured the infant’s interest by presenting them with an attractive toy (a glitter wand with a button-operated flashing colored light). The experimenter held up the wand, shared eye contact with the child, and said, “[Child’s name]. Don’t touch it. No.” The wand was then placed centrally on the table (within reaching distance of the infant), and the experimenter released the wand (sticky tack was secured underneath the wand to prevent the wand from rolling toward them). The experimenter then turned away from the infant and the wand for 30 s. If the infant had not touched the wand after 30 s, they were given the following prompts until the wand was touched: “It’s okay, you can touch it now” (after 30 s); “You can touch it” (after 35 s; experimenter points to the wand); “Let’s see what happens when we touch it” (after 40 s; at which point the experimenter demonstrates touching the wand). The task was terminated if the infant did not touch the wand after 45 s.

Prohibition performance was calculated as the latency in seconds to touch the wand (i.e., the time when the infant touched the wand minus the time when the wand was initially released by the experimenter), with a possible range of 0–30 s. Higher scores thus indicated better compliance with the prohibition. Four participants touched the wand after 30 s, and one participant never touched the wand. These five participants were assigned a latency to touch of 30 s. Double coding of 20 videos by two independent coders established excellent intercoder reliability (ICC (single measures) = 1.000, *p* < .001). A total of 22 participants did not complete the task (parental questionnaire responses only), resulting in a final sample of *n* = 141 with TP task data available.

This task is widely used as a measure of hot IC (Friedman et al., 2011; Hendry et al., 2021; McHarg et al., 2020a,), and although most infants fail to follow the prohibition in this task (e.g., 64.2 % in Devine et al., 2019), infants do vary in their performance, with some infants managing to wait the full delay.

#### 2.2.4 Early Executive Functions Questionnaire (EEFQ)

##### 2.2.4.1 Procedure

The Early Executive Functions Questionnaire (EEFQ; for details, see Hendry & Holmboe, 2021) is a 31-item parent-report measure comprised of four scales: (1) Inhibitory Control, (2) Flexibility, (3) Working Memory, and (4) Regulation. The EEFQ was completed online by parents prior to the lab visit. Previous research has established that this questionnaire has good psychometric properties, showing both convergence and divergence with existing measures of infant temperament as well as good test-retest reliability (see Hendry & Holmboe, 2021).

##### 2.2.4.2 Inhibitory Control Scale (EEFQ-IC)

The EEFQ-IC scale includes 8 items rated on a 7-point Likert scale. The first item involved parents playing a waiting game with their child to elicit IC in a semi-standardized context (for details, see Hendry & Holmboe, 2021). For the remaining 7 items, parents reported the frequency with which their child exhibited inhibitory behaviors in the past two weeks (e.g., “approach or reach for something that they have been repeatedly told not to touch (such as electrical sockets or the oven)”) on a 7-point scale (1 = *Never*, 7 = *Always*). Scores for the EEFQ-IC scale were calculated by computing the mean of all items in the scale (item scorings were reversed prior to averaging where appropriate, so that higher scores indicated higher IC). Data were included only where a minimum of 6 items (70 % of the scale) was completed. The scale demonstrated satisfactory internal consistency (α = .64). Nine respondents did not complete the minimum number of items to compute the scale mean, and three respondents did not complete the EEFQ-IC scale, resulting in a sample of *n* = 151 with EEFQ-IC data available.

##### 2.2.4.3 Regulation Scale (EEFQ-Reg)

Parents reported the frequency with which their child exhibited regulatory behaviors in the past two weeks (e.g., “return to being calm/happy within 3 min of a small frustration (e.g., not being able to do something)”) on a 7-point scale (1 = *Never*, 7 = *Always*). Scores for the 8-item Regulation scale (EEFQ-Reg) were calculated by computing the mean of all items in the scale (applying the 70 % minimum threshold). Item scorings were reversed prior to averaging where appropriate so that higher scores indicated higher regulation. The scale demonstrated good internal consistency (α = .76). Four respondents did not complete the minimum number of items (at least 6 items) to compute the scale mean, and three respondents did not complete the EEFQ-Reg scale, resulting in a sample of *n* = 156 with EEFQ-Reg data available.

##### 2.2.4.4 Cognitive Executive Function (EEFQ-CEF)

The Cognitive Executive Function (EEFQ-CEF) composite score encompasses Inhibitory Control (IC), Working Memory (WM), and Flexibility (FX) items. Scores for the 22-item EEFQ-CEF scale were calculated by computing the mean of all items from the EEFQ-IC, EEFQ-WM, and EEFQ-FX scales, where a minimum of 70 % of items across all three scales combined was completed. Higher EEFQ-CEF scores indicated better EF skills. The EEFQ-CEF composite demonstrated good internal consistency (α = .76). Ten respondents did not reach the minimum number of items (at least 15 items) to compute the EEFQ-CEF composite, and three respondents did not complete the EEFQ, resulting in a sample of *n* = 150 with EEFQ-CEF data available.

## 3 Results

### 3.1 Power analysis

A statistical power analysis was conducted using G*Power 3.1 (Erdfelder et al., 1996; Faul et al., 2007), based on the correlation reported in Barr et al. (2010) (*N* = 60) between total preschool household TV exposure and Behavior Rating Inventory of Executive Functioning–Preschool Version General Executive Function (BRIEF-P GEC) at age 4, *r* = .29 (for details, see Table 4, Barr et al., 2010). With a sample size of *n* = 150 for correlations involving touchscreen use (TUQ), our analysis had over 90 % power to detect an effect size of *r* = .29, with a two-tailed alpha level of .05.

### 3.2 Descriptive statistics

Descriptive statistics for all six measures (TUQ, ECITT, TP, EEFQ-IC, EEFQ-Reg, EEFQ-CEF) are presented in Table 2. Most measures showed a good range of scores except for the TP task, where 86 % of infants were performing at floor (latency under 5 s, see SM 6, Supplementary Fig. 1.3).

**Table 2:** Descriptive Statistics for All Measures.

	*n*	Mean	Median	*SD*	Min	Max
Amount of Touchscreen Exposure (TUQ)	150	2.09	2.00	0.85	1.00	5.29
Regulation (EEFQ-Reg)	156	5.90	6.00	0.74	2.43	7.00
Toy Prohibition (TP)	141	3.68	1.37	6.80	0.00	30.00
Inhibitory Control (EEFQ-IC)	151	3.55	3.50	0.76	1.71	5.71
Response Inhibition (ECITT)	128	0.59	0.58	0.36	0.00	1.55
Cognitive Executive Function (EEFQ-CEF)	148	3.89	3.82	0.60	2.62	5.57

*Note.* TUQ = Touchscreen Use Questionnaire. EEFQ-Reg = Regulation scale. EEFQ-IC = Inhibitory Control scale. ECITT = Early Childhood Inhibitory Touchscreen Task. EEFQ-CEF = Cognitive Executive Function score.

#### 3.2.1 Touchscreen Usage (TUQ)

On average, 79.3 % of 10-month-olds used touchscreen devices for video chatting with loved ones, 70.7 % for watching videos and looking at photos, 22.7 % for scrolling/swiping through photos/videos, 19.3 % for playing simple games, and 8 % for doing drawings or scribbles (for descriptive statistics for each item, see SM 2). Note that these percentages refer to infants who have had *some* exposure by 10-months (for each type of content), compared to never having engaged with that type of content. In other words, these percentages were calculated as [100 % – % of participants who have answered “*Never*”] for any given touchscreen activity.

#### 3.2.2 Response Inhibition (ECITT)

A paired-samples *t*-test revealed that infants have a significantly higher level of accuracy on prepotent trials (*M* = .88, *SD* = .11) compared to inhibitory trials (*M* = .51, *SD* = .30); *t*(127) = −13.26, *p* < .001, *d* = 1.63 (note that this analysis is also reported in Fiske et al., 2021). The inhibitory score is a reversed version of the adjusted AccD (the difference between performance on the inhibitory and prepotent trials divided by performance on the prepotent trials), such that a *higher score* indicates a *higher level* of IC. An alternative index is the reversed unadjusted AccD score, a simple measure of inhibitory performance that does not take performance on the prepotent trials into account. Results using the AccD were highly consistent with those reported using the inhibitory score (for correlation analyses using the AccD instead of the inhibitory score, see SM 7, Supplementary Table 6).

### 3.3 Correlation analyses

The Shapiro-Wilk test, histograms, and normal Q-Q plots revealed that all measures except EEFQ-IC and EEFQ-CEF violated the assumption of normality (see SM 6). Therefore, the Spearman rank-order correlation coefficient was used to assess bivariate associations between touchscreen exposure and the five measures for IC (broadly spanning hot and cool indices of IC, as well as a general cognitive EF factor): (1) EEFQ-Reg, (2) TP, (3) EEFQ-IC, (4) ECITT, and (5) EEFQ-CEF. The bivariate correlations are presented in Table 3. No significant association was found between the amount of touchscreen exposure and IC performance on the hot and cool measures of IC. A nominally significant positive association was found between Amount of Touchscreen Exposure and EEFQ-IC (*p* = .031) and EEFQ-CEF (*p* = .036, see Table 3). However, these associations did not survive correction for multiple comparisons using the Benjamini and Hochberg (1995) procedure.

**Table 3:** Correlations Between Amount of Touchscreen Exposure and IC and EF Measures.

	*n*	1	2	3	4	5	6
1. Amount of Touchscreen Exposure (TUQ)	150	–					
2. Regulation (EEFQ-Reg)	156	−.13	–				
3. Toy Prohibition (TP)	141	.02	.10	–			
4. Inhibitory Control (EEFQ-IC)	151	.18*	−.05	.15	–		
5. Response Inhibition (ECITT)	128	−.01	.08	.15	.03	–	
6. Cognitive Executive Function (EEFQ-CEF)	148	.18*	−.11	.02	.75**	−.05	–

*Note.* Spearman’s Rho correlation coefficients are reported here. TUQ = Touchscreen Use Questionnaire. EEFQ-Reg = Regulation scale. EEFQ-IC = Inhibitory Control scale. ECITT = Early Childhood Inhibitory Touchscreen Task. EEFQ-CEF = Cognitive Executive Function score.

**p < .01, *p < .05, two-tailed, uncorrected for multiple comparisons.

**Table 4:** Hierarchical Multiple Regression for the Amount of Touchscreen Exposure Scale and IC Measures.

	EEFQ-Reg		TP		EEFQ-IC		ECITT		EEFQ-CEF	
	1	2	1	2	1	2	1	2	1	2
***R^2^***	.05	.05	.03	.04	.08	.10	.02	.03	.09	.12
***ΔR^2^***	.05	.01	.03	.00	.08	.02	.02	.00	.09	.03
***ΔF***	1.04	1.02	0.71	0.61	1.80	1.91	0.44	0.38	2.01	2.45*

*Note.* EEFQ-Reg = Regulation scale. EEFQ-IC = Inhibitory Control scale. ECITT = Early Childhood Inhibitory Touchscreen Task. EEFQ-CEF = Cognitive Executive Function score. Step 1 = Infant’s gender, infant’s age, mother’s age, father’s age, maternal education (in years), paternal education (in years). Step 2 = Amount of Touchscreen Exposure.

*p < .05, two-tailed.

Given that previous studies have found no effects of screen exposure on Flexibility and Working Memory, a full correlation matrix, including correlations between the touchscreen scales, IC measures, the Flexibility (EEFQ-FX) scale, and Working Memory (EEFQ-WM) scale, was generated to explore whether this was also the case in the current data set (see SM 8). Note that the EEFQ-FX and EEFQ-WM scale scores were not included in the primary analyses due to weak internal consistency at the scale level (EEFQ-FX: α = .55; EEFQ-WM: α = .58). While no significant association was found between touchscreen exposure and EEFQ-WM, a nominally significant positive association was found for EEFQ-FX (*r*_s_ = .18, *p* = .037), although this did not survive correction using the Benjamini and Hochberg (1995) procedure (see SM 8, Supplementary Table 7). For associations between the individual touchscreen scale items and the IC/EF measures, see SM 3, Supplementary Table 3.2.

### 3.4 Hierarchical multiple regression

#### 3.4.1 Amount of touchscreen exposure

To further assess the effects of touchscreen exposure, above and beyond sociodemographic characteristics that may affect both IC performance and touchscreen exposure, hierarchical multiple regression was performed for each IC measure (the outcome variable), resulting in a total of five models. For the predictors at Step 1, background variables that most closely mapped onto the variables controlled for in McHarg et al.’s (2020a) study were added. This included infant’s gender, infant’s age, mother’s age, father’s age, maternal education in years (a common proxy for SES), and paternal education in years. The Amount of Touchscreen Exposure scale was entered at Step 2 to determine whether the amount of touchscreen exposure accounts for variance in IC performance after controlling for these sociodemographic predictors, resulting in a 2-step model for each IC measure. The *R*, Δ*R^2^*, and *F* change values for Steps 1 and 2 are presented in Table 4 (for full tables, see SM 9).

Before a hierarchical multiple regression was performed, preliminary analyses (i.e., Cook’s and Mahalanobis distance, tolerance and VIF statistics, plots of standardized and predicted residuals) were conducted to ensure no violation of the assumption of normality, linearity, multicollinearity, and homoscedasticity (see SM 7). All outcome variables met the assumptions except for TP, where the residuals violated the assumptions of normality. Closer inspection of the outliers, however, suggested that it did not significantly influence the model as Cook’s distance values were below 1 (Tabachnick & Fidell, 2013). A regression model for TP was therefore performed considering that other assumptions were satisfied.

As shown in Table 4, when sociodemographic variables were accounted for, the amount of touchscreen exposure was not significantly associated with any of our primary IC measures. However, there was a weak association between touchscreen exposure and EEFQ-CEF once sociodemographic variables were accounted for: introducing the Amount of Touchscreen Exposure scale at Step 2, the model as a whole explained 12 % of the variance in EEFQ-CEF, *F*(7, 125) = 2.45, *p* = .022. After controlling for Step 1 variables, Amount of Touchscreen Exposure explained an additional 3.3 % of the variance in EEFQ-CEF scores, and this change was significant, *F*(1, 125) = 4.73, *p* = .032 (Table 4).

#### 3.4.2 Age of initial touchscreen exposure

As the Age of Initial Touchscreen Exposure scale did not form a coherent scale (see Section 2.2.1.3), each item from the Age of Initial Touchscreen Exposure scale was added independently at Step 2 to determine whether the age of initial touchscreen exposure (at the item level) accounts for variance in IC and general EF performance. To ensure that any effects come from the single item under examination, Step 1 included all the same sociodemographic variables (infant’s gender, infant’s age, mother’s age, father’s age, maternal education (in years), and paternal education (in years) as well as the four items from the Age of Initial Touchscreen Exposure scale not being examined (e.g., (a) watch videos/look at photos, (b) scroll/swipe through photos or videos, (c) have video calls with loved ones, (d) play simple games). The single item being explored was entered at Step 2 (e.g., (e) do drawings/scribbles). This was repeated for the *five* items in the scale, and each item was analyzed *five* times (i.e., for each outcome measure), resulting in a total of 25 models. No significant effects were found between the age at which an infant initially engages with touchscreens and their IC or general EF performance. The *R*, Δ*R^2^*, and *F* change values for Steps 1 and 2 are reported in Table 5 (for full tables including other values, see SM 9).

**Table 5:** Hierarchical Multiple Regression for Items on the Amount of Touchscreen Exposure Scale.

		Predictors									
Outcomes		Watch/look at videos/photos		Scroll/swipe		Video chat		Playing games		Drawing/scribbling	
		1	2	1	2	1	2	1	2	1	2
	***R^2^***	.07	.08	.08	.08	.07	.08	.06	.08	.08	.08
**EEFQ-Reg**	**Δ*R^2^***	.07	.00	.08	.00	.07	.01	.06	.02	.08	.00
	**Δ*F***	0.98	0.28	0.99	0.22	0.94	0.70	0.74	2.54	1.00	0.10

	***R^2^***	.06	.07	.07	.07	.06	.07	.05	.07	.06	.07
**TP**	**Δ*R^2^***	.06	.01	.07	.00	.06	.01	.05	.02	.06	.01
	**Δ*F***	0.76	0.98	0.07	0.00	0.77	0.87	0.66	0.66	0.76	0.98

	***R^2^***	.09	.11	.10	.11	.11	.11	.10	.11	.10	.11
**EEFQ-IC**	**Δ*R^2^***	.09	.02	.10	.00	.11	.00	.10	.00	.10	.01
	**Δ*F***	1.15	2.59	1.41	0.23	1.43	0.00	1.42	0.12	1.35	0.74

	***R^2^***	.07	.08	.04	.08	.08	.08	.05	.08	.07	.08
**ECITT**	**Δ*R^2^***	.07	.01	.04	.03	.08	.00	.05	.02	.07	.00
	**Δ*F***	0.77	0.61	0.43	3.80	0.84	0.02	0.56	2.63	0.82	0.15

	***R^2^***	.09	.10	.10	.10	.10	.10	.10	.10	.10	.10
**EEFQ-CEF**	**Δ*R^2^***	.09	.01	.10	.00	.10	.00	.10	.00	.10	.00
	**Δ*F***	1.24	1.18	1.37	0.01	1.37	0.02	1.36	0.06	1.37	0.04

*Note.* EEFQ-Reg = Regulation scale. EEFQ-IC = Inhibitory Control scale. ECITT = Early Childhood Inhibitory Touchscreen Task. EEFQ-CEF = Cognitive Executive Function score. Step 1 = Infant’s gender, infant’s age, mother’s age, father’s age, maternal education (in years), paternal education (in years), Amount of Touchscreen Exposure items (four items). Step 2 = Amount of Touchscreen Exposure item (one item).

## 4 Discussion

This study aimed to explore the effects of touchscreen exposure on both hot and cool IC development in 10-month-old infants, namely skills relating to Regulation (EEFQ-Reg), Prohibition (TP), Inhibitory Control (EEFQ-IC), and Response Inhibition (ECITT). A secondary broader EF construct, Cognitive Executive Function (EEFQ-CEF), was also examined to determine whether these effects were specific to IC or related to EF more generally. After correcting for multiple comparisons, correlation analyses revealed no association between infant touchscreen exposure and any of the four indices of IC or EEFQ-CEF. When sociodemographic variables (infant’s gender, infant’s age, mother’s age, father’s age, maternal education, and paternal education) were controlled for, a significant *positive* association was found between the amount of touchscreen exposure and EEFQ-CEF. No significant effects were found for the age at which infants are initially exposed to touchscreens and their subsequent IC and EEFQ-CEF.

The present study did not replicate prior findings which have indicated a *negative* association between screen exposure at age 2 and EF performance at age 3 when assessed using a composite EF score (McHarg et al., 2020b). The adverse effects of screen use for IC (specifically hot IC) were also stronger compared to other EF domains (working memory and cognitive flexibility) when screen exposure occurred during infancy (McHarg et al., 2020a). There are several possible reasons for this discrepancy. Firstly, McHarg et al. (2020a, 2020b) have primarily focused on passive forms of screen engagement (i.e., viewing content on TVs and touchscreens), whereas the current study focused on passive, active, and social forms of *touchscreen* engagement. The issue of background screen exposure, namely having the TV or attention-grabbing device playing in the background, may be more detrimental for EF development as this is likely to disrupt infants’ attentional processes (Ribner et al., 2017) and their opportunity to practice regulation (Coyne et al., 2021).

Another important consideration relates to the interactive aspect of touchscreens. Previous literature found no association between EF skills and screen use amongst preschool children when this included TVs and touchscreens (Jusienė et al., 2020). Similarly, IC performance was impaired only after children viewed a cartoon, but not when they interacted with these events via a touchscreen app (Huber et al., 2018; Li et al., 2018). The process of screen interaction may thus have mitigated the negative effects typically associated with screen use. We also note that a common use of touchscreens was to have video calls with loved ones (79 % of our sample did this at least occasionally, and 21 % did this 2–4 times per week, see SM 2 for details). Given that higher-order cognitive processes often develop in contexts rich in interpersonal interactions and when children engage in age-appropriate activities (e.g., imaginative play; Bernier et al., 2010; Carlson & White, 2013), the social contingency involved in video chatting may have buffered against potential negative effects of touchscreen use by promoting the development of higher quality social and parent-child interactions (Roseberry et al., 2013). Together, the interactivity and social contingency aspects of touchscreens may partly explain the positive association between touchscreen exposure and EEFQ-CEF.

Another explanation for the discrepant findings pertains to the different methods of assessing IC or EF. Although the prohibition task used to assess IC was the same task as used in McHarg et al.’s (2020a) study, the composite EF score used in McHarg et al.’s (2020b) later study was based on laboratory tasks, whereas the EEFQ-CEF score in the current study was based on parental report. The EEFQ-CEF score may be capturing a different aspect of EF, namely the EF skills needed for performing tasks in daily life rather than in controlled and unfamiliar settings. While we found a positive association between early touchscreen exposure and EEFQ-CEF, this effect was small (β = .19, see SM 9 for details) and must thus be interpreted cautiously.

### 4.1 Strengths and limitations

Several limitations should be noted. Firstly, the current sample was not representative of the general population, as indicated by the skew towards highly educated, predominantly White parents (see Table 1). Further research is needed to understand how screen use impacts EF development in contexts involving socioeconomic disadvantage, where screen use may be higher due to the influence of external factors, such as attitudinal differences (Njoroge et al., 2013), additional constraints on parents’ time and well-being (Ribner et al., 2017), and limited availability of alternative activities and space to play (Martin-Biggers et al., 2015).

Another limitation was that the purpose of screen use (e.g., education, pacifying, seeing loved ones) and parental co-viewing was not assessed. While video chatting may facilitate more positive social engagement due to higher parental involvement (McClure et al., 2015), using screens to “manage” emotional outbursts may hinder EF (Coyne et al., 2021). However, the cross-sectional nature of our analyses prevents us from establishing the direction of causality, thus it is unclear whether bi-directional relationships are in play. For example, the association between touchscreen exposure and EF could be driven by infants who have better EF to begin with, and are therefore less likely to be given a screen device to manage their emotions. While the present study did assess passive, active, and social touchscreen engagement, it was not feasible to create subscales for all these facets with the current number of items; for instance, social touchscreen engagement consisted of only one item (i.e., video chatting). Future studies should explore a broader range of passive, active, and social touchscreen activities during infancy and assess the amount and nature of parental co-use of screens to ascertain their role in EF development.

Furthermore, none of the participants were particularly high touchscreen users (see Table 2). The modest use of touchscreens may partly explain the lack of a negative association between touchscreen exposure and IC/EF, such that adverse effects may only appear through excessive use. Alternatively, adverse outcomes of early screen exposure may appear later in development rather than immediately. Indeed, while screen time at 24-months predicted lower EF performance a year later, concurrent screen time and EF were unrelated when assessed at 36-months (McHarg et al., 2020b). Previous studies reporting negative effects of infant screen exposure on IC specifically have also been longitudinal in design (e.g., Barr et al., 2010; McHarg et al., 2020a), thus allowing the prediction of later outcomes. Future research should adopt longitudinal analyses to ascertain whether the effects of touchscreen exposure occur gradually rather than immediately.

Finally, while the EEFQ-CEF composite used in this study was informed by a factor analysis of EEFQ data in previous work (Hendry & Holmboe, 2021), the sample size of the current study did not allow for a well-powered factor analysis, given our particular data structure (Tabachnick & Fidell, 2013). Further, as correlations between the individual indicators of IC were generally low and non-significant (see Table 3) – a finding commonly observed in EF research (e.g., Huizinga et al., 2006) – we were not able to reduce our data down to a single indicator of IC or EF. Instead, we used separate correlational analyses and hierarchical regression analyses for each IC indicator. Although a limitation of this approach was the number of multiple comparisons, a strength was that we were able to establish whether conclusions differ by measurement type and by hot versus cool EF. Notably, no negative association was found between touchscreen exposure and IC regardless of assessment and EF type, although a positive association was found between touchscreen exposure and parent-reported composite EF score.

### 4.2 Implications

In light of the COVID-19 pandemic, the present findings have an especially important implication since screen use has proliferated amongst young children in the UK and many other countries globally (Bergmann et al., 2021). A benefit of screen use during the pandemic has been enabling young children to remain connected with loved ones. Although the AAP (1999, 2016) guidelines were revised in 2016 to remove video calls from their recommended media restrictions for children under 2, these findings urge policymakers and researchers to re-evaluate the WHO guidelines for children under age 2 to provide more specific advice on the benefits and risks of touchscreen use more generally in infancy, taking into account the findings of this study indicating benign effects, alongside other studies indicating possible negative effects on EF development (e.g., Lawrence et al., 2020; McHarg et al., 2020a, 2020b). It is important to note, however, that the strict WHO (2019) guidelines on infants might not be based on the negative effects of screens on cognition but rather on their physical health. Nonetheless, these guidelines hold important implications as they may influence the amount of screen time parents allow for their children.

### 4.3 Conclusion

Overall, the present study found no negative association between touchscreen exposure during infancy and hot and cool IC development. When sociodemographic variables were accounted for, greater touchscreen exposure showed a small *positive* association with parent-reported cognitive EF at 10-months of age. Contrary to the mainstream and often negative narrative surrounding touchscreen usage (Davis, 2018; Ortiz, 2018; Weale, 2016), there was no indication that moderate early touchscreen exposure is linked to poor IC or general EF skills in infancy, potentially reducing the “moral panic” experienced by parents regarding early touchscreen exposure (Drotner, 2013). Exploring the benefits of passive, active, and social touchscreen engagement for infant IC and EF development is an important avenue for future research. The current study provides a springboard for ongoing and future follow-up assessments to establish the potential impacts of early touchscreen usage and whether these last beyond the first year.

## Funding

This work was supported by a Career Development Award from the 10.13039/501100007155Medical Research Council [MR/N008626/1] to K. Holmboe. A. Hendry was supported by an 10.13039/501100000269Economic and Social Research Council Postdoctoral Fellowship [ES/S011730/1] and by the Scott Family Junior Research Fellowship in Autism (University College Oxford).

## Data statement

The data reported in this article are openly available in anonymized form via OSF (https://osf.io/w8gs4/), except where participants have opted out of data sharing on the study consent form.

## CRediT authorship contribution statement

**Katie Y.K. Lui:** Conceptualization, Formal analysis, Investigation, Methodology, Writing - original draft, Writing - review & editing. **Alexandra Hendry:** Conceptualization, Formal analysis, Funding acquisition, Investigation, Methodology, Writing - review & editing. **Abigail Fiske:** Methodology, Investigation, Supervision, Writing - review & editing. **Henrik Dvergsdal:** Software, Writing - review & editing. **Karla Holmboe:** Conceptualization, Data curation, Formal analysis, Funding acquisition, Investigation, Methodology, Project administration, Resources, Supervision, Validation, Writing - review & editing.

## Declaration of Competing Interest

The authors declare that they have no known competing financial interests or personal relationships that could have appeared to influence the work reported in this paper.
