# Supplementary material for: Associations between touchscreen exposure and hot and cool inhibitory control in 10-month-old infants
Source: Infant Behav Dev. 2021 Nov;65:101649. doi: 10.1016/j.infbeh.2021.101649 (PMC8641060; doi:10.1016/j.infbeh.2021.101649)
Supplement: Supplementary file 1 [file mmc1.docx]

**Associations between touchscreen exposure and hot and cool inhibitory control in 10-month-old infants**

**Supplementary Materials 1: Touchscreen Use Questionnaire (TUQ)**
