# Supplementary material for: Associations between touchscreen exposure and hot and cool inhibitory control in 10-month-old infants
Source: Infant Behav Dev. 2021 Nov;65:101649. doi: 10.1016/j.infbeh.2021.101649 (PMC8641060; doi:10.1016/j.infbeh.2021.101649)
Supplement: Supplementary file 2 [file mmc2.docx]

**Associations between touchscreen exposure and hot and cool inhibitory control in 10-month-old infants**

**Supplementary Materials 2: Descriptive Statistics for Touchscreen Use Questionnaire (TUQ)**

# Supplementary Table 1.1

*Frequency (%) and Mean Score of Participants Performing Each Touchscreen Activity on the Amount of Touchscreen Exposure Scale*

|  | **%** | | | | | | | **Mean score (SD)** |
| --- | --- | --- | --- | --- | --- | --- | --- | --- |
|  | **1** | **2** | **3** | **4** | **5** | **6** | **7** |  |
|  | ***Never*** | ***Once per month or less*** | ***2–4 times per month*** | ***5–8 times per month*** | ***More than twice per wee*k** | ***2–4 times per week*** | ***Most days*** |  |
| *Touchscreen activities* |  |  |  |  |  |  |  |  |
| 1. Watch videos/look at photos 2. Scroll/swipe through photos/videos 3. Have video calls with loved ones 4. Play simple games 5. Do drawings or scribbles | 29.3  77.3  20.7  80.7  92.0 | 14.7  7.3  16.0  8.0  4.7 | 14.0  4.0  14.0  3.3  1.3 | 12.7  3.3  12.7  2.0  0.0 | 8.7  4.0  4.0  2.7  2.0 | 13.3  2.0  20.7  2.0  0.0 | 7.3  2.0  12.0  1.3  0.0 | 3.26 (2.03)  1.63 (1.42)  3.73 (2.13)  1.49 (1.25)  1.15 (0.63) |
|  | ***> 5 mins*** | ***5–20***  ***mins*** | ***20–60***  ***mins*** | ***1–2 hours*** | ***2–4***  ***hours*** | ***4–6 hours*** | ***≥ 7***  ***hours*** | **Mean (SD)** |
| *Total duration using touchscreen*   1. Duration of looking only 2. Duration of interacting only | 44.0  76.0 | 30.0  16.7 | 15.3  3.3 | 6.0  2.7 | 3.3  0.7 | 1.3  0.7 | 0.0  0.0 | 1.99 (1.16)  1.37 (0.82) |

*Note. n* = 150.

# Supplementary Table 1.2

*Frequency (%) and Mean Score of Participants Performing Each Touchscreen Activity on the Age of Initial Touchscreen Exposure Scale*

|  | **%** | | | | **Mean score (SD)** |
| --- | --- | --- | --- | --- | --- |
|  | **1** | **2** | **3** | **4** |  |
|  | ***Before 6 months*** | ***6–9 months*** | ***9–12 months*** | ***Has not done this yet*** |  |
| *Touchscreen activities* |  |  |  |  |  |
| 1. Watch videos/look at photos | 38.7 | 25.3 | 6.0 | 30.0 | 2.73 (1.26) |
| 1. Scroll/swipe through photos/videos | 4.0 | 15.3 | 5.3 | 75.3 | 1.48 (0.90) |
| 1. Have video calls with loved ones | 63.3 | 17.3 | 2.7 | 16.7 | 3.27 (1.12) |
| 1. Play simple games | 4.0 | 10.0 | 4.0 | 82.0 | 1.36 (0.82) |
| 1. Do drawings or scribbles | 2.0 | 5.3 | 1.3 | 91.3 | 1.18 (0.61) |

*Note. n* = 150.

**Supplementary Table 2**

*Percentage (%) and Mean Score of Parental Ratings of Participants’ Level of Enjoyment of Using Touchscreens*

| **%** | | | | | | **Mean score**  **(SD)** |
| --- | --- | --- | --- | --- | --- | --- |
| **1** | **2** | **3** | **4** | **5** | **6** |  |
| **Does not have the opportunity at all** | **Not at all** | **A little** | **A moderate amount** | **A lot** | **A great deal** |  |
| 50.7 | 0.7 | 13.3 | 13.3 | 12.0 | 10.0 | 2.65 (1.85) |

*Note. n* = 150.
