# Supplementary material for: Associations between touchscreen exposure and hot and cool inhibitory control in 10-month-old infants
Source: Infant Behav Dev. 2021 Nov;65:101649. doi: 10.1016/j.infbeh.2021.101649 (PMC8641060; doi:10.1016/j.infbeh.2021.101649)
Supplement: Supplementary file 3 [file mmc3.docx]

**Associations between touchscreen exposure and hot and cool inhibitory control in 10-month-old infants**

# Supplementary Materials 3: Item Level Analysis of Touchscreen Use Questionnaire (TUQ)

**Supplementary Table 3.1**

*Correlation Matrix of Individual Touchscreen Items on the Touchscreen Use Questionnaire (TUQ)*

|  |  | **1** | **2** | **3** | **4** | **5** | **6** | **7** | **8** | **9** | **10** | **11** | **12** |
| --- | --- | --- | --- | --- | --- | --- | --- | --- | --- | --- | --- | --- | --- |
| **Amount of Exposure** | 1. Watch videos | **–** |  |  |  |  |  |  |  |  |  |  |  |
|  | 1. Scroll/swipe photos | .376^**^ | – |  |  |  |  |  |  |  |  |  |  |
|  | 1. Video call loved ones | .301^**^ | .185^*^ | – |  |  |  |  |  |  |  |  |  |
|  | 1. Play simple games | .202^*^ | .214^**^ | .383^**^ | – |  |  |  |  |  |  |  |  |
|  | 1. Do drawings/scribbles | .110 | .073 | .198^*^ | .110 | – |  |  |  |  |  |  |  |
|  | 1. Duration of looking | .504^**^ | .198^*^ | .425^**^ | .156 | .232^**^ | – |  |  |  |  |  |  |
|  | 1. Duration of interacting | .326^**^ | .428^**^ | .046 | .369^**^ | .086 | .264^**^ | – |  |  |  |  |  |
| **Age of Initial Exposure** | 1. Watch videos | .550^**^ | .171^*^ | .255^**^ | .308^**^ | .041 | .289^**^ | .275^**^ | – |  |  |  |  |
|  | 1. Scroll/swipe photos | .332^**^ | .705^**^ | .136 | .231^**^ | .024 | .177^*^ | .397^**^ | .225^**^ | – |  |  |  |
|  | 1. Video call loved ones | .217^**^ | .055 | .623^**^ | .192^*^ | .179^*^ | .336^**^ | –.054 | .296^**^ | .068 | – |  |  |
|  | 1. Play simple games | .144 | .321^**^ | .339^**^ | .764^**^ | .271^**^ | .106 | .302^**^ | .199^*^ | .264^**^ | .161^*^ | – |  |
|  | 1. Do drawings/scribbles | .020 | .053 | .140 | .085 | .769^**^ | .154 | –.004 | –.015 | .005 | .189^*^ | .250^**^ | – |

*Note. n* = 150. Spearman's Rho correlation coefficients are reported here.

*^**^p* < .01, ^*^*p* < .05, two–tailed, uncorrected for multiple comparisons.

# Supplementary Table 3.2

*Correlation Matrix of IC and EF Measures with Individual Items on the Touchscreen Use Questionnaire (TUQ)*

|  |  | ***n*** | **1** | **2** | **3** | **4** | **5** | **6** | **7** | **8** | **9** | **10** | **11** | **12** | **13** | **14** | **15** | **16** | **17** |
| --- | --- | --- | --- | --- | --- | --- | --- | --- | --- | --- | --- | --- | --- | --- | --- | --- | --- | --- | --- |
| **EEFQ–Reg** | 1. Regulation | 156 | **–** |  |  |  |  |  |  |  |  |  |  |  |  |  |  |  |  |
| **TP** | 1. Toy Prohibition | 141 | .097 | – |  |  |  |  |  |  |  |  |  |  |  |  |  |  |  |
| **EEFQ–IC** | 1. Inhibitory Control | 151 | –.051 | .149 | – |  |  |  |  |  |  |  |  |  |  |  |  |  |  |
| **ECITT** | 1. Response Inhibition | 128 | .084 | .146 | .033 | – |  |  |  |  |  |  |  |  |  |  |  |  |  |
| **EEFQ–CEF** | 1. Cognitive Executive Function | 148 | –.112 | .020 | .749^**^ | –.049 | – |  |  |  |  |  |  |  |  |  |  |  |  |
| **Amount of Exposure** | 1. Watch videos | 150 | .003 | –.009 | .125 | –.003 | .052 | – |  |  |  |  |  |  |  |  |  |  |  |
|  | 1. Scroll/swipe photos | 150 | –.116 | .098 | .011 | –.052 | .040 | .376^**^ | – |  |  |  |  |  |  |  |  |  |  |
|  | 1. Video call loved ones | 150 | –.017 | .024 | .149 | .057 | .217^*^ | .301^**^ | .185^*^ | – |  |  |  |  |  |  |  |  |  |
|  | 1. Play simple games | 150 | –.097 | .117 | .106 | .118 | .166 | .202^*^ | .214^**^ | .383^**^ | – |  |  |  |  |  |  |  |  |
|  | 1. Do drawings/scribbles | 150 | –.036 | –.015 | .057 | .056 | .025 | .110 | .073 | .198^*^ | .110 | – |  |  |  |  |  |  |  |
|  | 1. Duration of looking | 150 | –.163^*^ | –.069 | .085 | .002 | .123 | .504^**^ | .198^*^ | .425^**^ | .156 | .232^**^ | – |  |  |  |  |  |  |
|  | 1. Duration of interacting | 150 | –.171^*^ | .025 | .120 | –.102 | .103 | .326^**^ | .428^**^ | .046 | .369^**^ | .086 | .264^**^ | – |  |  |  |  |  |
| **Age of Initial Exposure** | 1. Watch videos | 150 | –.062 | –.048 | .150 | .044 | .122 | .550^**^ | .171^*^ | .255^**^ | .308^**^ | .041 | .289^**^ | .275^**^ | – |  |  |  |  |
|  | 1. Scroll/swipe photos | 150 | –.059 | –.099 | .026 | –.138 | .074 | .332^**^ | .705^**^ | .136 | .231^**^ | .024 | .177^*^ | .397^**^ | .225^**^ | – |  |  |  |
|  | 1. Video call loved ones | 150 | .007 | .070 | .023 | .027 | .011 | .217^**^ | .055 | .623^**^ | .192^*^ | .179^*^ | .336^**^ | –.054 | .296^**^ | .068 | – |  |  |
|  | 1. Play simple games | 150 | –.139 | .092 | .061 | .048 | .127 | .144 | .321^**^ | .339^**^ | .764^**^ | .271^**^ | .106 | .302^**^ | .199^*^ | .264^**^ | .161^*^ | – |  |
|  | 1. Do drawings/scribbles | 150 | –.012 | .088 | .070 | .014 | –.004 | .020 | .053 | .140 | .085 | .769^**^ | .154 | –.004 | –.015 | .005 | .189^*^ | .250^**^ | – |

*Note.* Spearman's Rho correlation coefficients are reported here. EEFQ–Reg = Regulation scale. EEFQ–IC = Inhibitory Control scale. ECITT = Early Childhood Inhibitory Touchscreen Task. EEFQ–CEF = Cognitive Executive Function score.
*^**^p* < .01, ^*^*p* < .05, two–tailed, uncorrected for multiple comparisons.
