# Supplementary material for: Associations between touchscreen exposure and hot and cool inhibitory control in 10-month-old infants
Source: Infant Behav Dev. 2021 Nov;65:101649. doi: 10.1016/j.infbeh.2021.101649 (PMC8641060; doi:10.1016/j.infbeh.2021.101649)

**Associations between touchscreen exposure and hot and cool inhibitory control in 10-month-old infants**

**Supplementary Materials 4: 10m ECITT Coding Protocol**

Coding protocol

*Note that if the infant is incorrect the trial does not end until the happy face is hit…. this can take some time. Only start coding accuracy and validity again for the next trial (i.e. after a cartoon has been played).*

**To view the template and an example of the ECITT coding, see SM 3. Note that all identifying information has been removed.**

**Accuracy** (column X) is either 0 (incorrect) or 1 (correct). Accuracy is taken from the first response (defined as bash, touch, tap, scratch etc.) to or near a target (blue button with or without happy face)

- Note: the response could count as dragging their hand over the screen, scratching/scraping the screen, bashing with their full hand consistently at one side of the screen.
- If the infant touches the middle, top, or bottom OUTSIDE of the target area this is not considered a RESPONSE. CODE NEXT RESPONSE.
- **If the child taps outside a hit area and then seems to change direction, we have to take the first response from the first tap within the hit area.**
- When the infant touches the wrong side for balance but is clearly looking at the correct side and touches it on their next response, code their second response as opposed to their first touch (i.e. the balancing touch) and overwrite software accuracy.

**Validity**: 0 = invalid. 1 = valid. If invalid specify reason code in column Z.

- IMPORTANT: If child has finger on one of the buttons (or within the touch sensitive area) at stim onset, the trial is always invalid.
- **First trial is ALWAYS invalid.** This is because the experimenter cues the correct side on the first trial. (In some older videos, this did not happen, but we code ALL first trials as invalid for consistency).
- **Validity (based on RT):** Paste in the formula =IF(S2<300,0, 1) and drag this down the column. Check the results make sense - trials with a respTime less than 300 should show as 0 in the valid column. All other trials should be 1.

**Reason invalid**

**1 =** Taps with 2 hands simultaneously (intentional response)

- Use this sparingly, often one hand does slightly lead and the other one is just being used for balance. Go with what is touched first and don’t use invalidity code.
- Code accuracy as per the software.
- If an intentional, 2 hand response is made where both hands simultaneously touch the screen, then it is invalid. This is because it is impossible to tell what response the participant wanted to make. Use only when very clear that response was intentional and simulatanious.

**2** = First tap was not registered and then changed to a different side;

- **Do not use as invalidity code** – instead code the accuracy of the first tap and treat as valid (regardless of whether it agrees with the software accuracy code or not)

**3** = Still had hand in position from previous tap (or already reaching before the new set of stimuli had been shown), so response probably not intentional.

- Note that this will often be accompanied by a short RT: Only exclude as invalid if <300MS

**4** = Infant clearly not attending and tapped by **accident.**

- Examples of accidental tapping include but are not limited to: looking at E1 or mum when making a response, touched the screen when bashing and fussing, fidgeting with the tape around the tablet, not looking at the screen when responding, touching the screen to balance, catching the screen with hand when moving tablet or when E1 moves tablet.
- At 10m – this often happens when infant is trying to push/pull tablet away and then accidentally draws hand across screen – thus there is a response, but it's not deliberately directed to a stimulus
- Bimanual bashing (often multiple times), if child is bashing/touching screen while not looking (e.g., head turned at least 90% away from the screen) should be coded as invalid (accident).

**5** = Parent interference

- Points/nudges or otherwise indicates which one to touch

**6** = Researcher pressed

- This may occur early if the researcher thought they needed another cue, or by accident later in the task

**7** = Reaching behaviour obscured

- (e.g. child stands on mum’s lap, so screen and reach are just out of range of video).
- ONLY USE WHEN RESPONSE IS STILL UNCLEAR ON CAMERA 2 or 3 – often you can use Camera 2 or 3 to acknowledge the looking behaviour/motor response of the infant to guide judgement. If infant is clearly orienting towards one side of the tablet, use this to deduce whether they made a correct / incorrect response

**8** = Used parent’s hand – This is unlikely to occur

**9** = Experimenter interference

- Experimenter directly points to or says which location is correct/incorrect (includes initial prompt on the first trial)
- Experimenter stops incorrect response in progress (child about to make response) but stops because the experimenter indicates that this is incorrect).

**10** = On first prepotent trial infant tapped the opposite side so the researcher then restarted with the opposite side marked as prepotent

**11 =** Experimenter changes prepotent sides halfway through the trial – trial numbers in column M will reset to number 1

**12 =** If child has finger/hand/both hands on one, or both, of the stimuli (or within a touch sensitive area) **at or before stimulus onset** then the trial is **always** invalid. Override software when needed.

**13 =** Child touches middle of screen. This is a clear intentional response (e.g., not an accident) but it is impossible to tell which stimuli the child intended to touch. Software will make its own decision. Code accuracy with software, but invalid.

**14** = Infant responds using area of body that is not their hands (e.g. nose, forehead, foot, mouth, elbow etc.)

**Notes on Validity**

- It’s okay for the child to turn away for any duration, including at the beginning of trials (e.g., at stim onset).
- Trials with long RTs will be excluded in Step 2 for RT analyses, but for Accuracy it is okay to include these trials.
- If the infant gets stuck on an inhibitory trial (i.e. they make an incorrect response, but they can’t/won’t move on), the research may tap the correct side to trigger the cartoon and the next trial. This is fine as this response doesn’t affect the accuracy data.
- If a trial is invalid, still code the accuracy as if it had been valid (for example if the infant inadvertently swipes the tablet, record whether it was on the correct side or not)

**Reason Overriding Data**

*NB: Child’s actual behaviour observed on the video will ALWAYS override software. It is sometimes the case that the software does not detect the child’s first response to a target area – mostly in this case (if accurate), the experimenter will touch for the child. Always keep comments in the ‘Notes’ column when this happens*

In cases where you need to manually override the software, record a ‘Y’ for yes in column ‘AD’

Provide a reason for overwriting the software in column ‘AE’

Highlight where you have overwritten in your own coding columns and also in ‘accu’ column on software (Column P) - so that we can check this with another coder.

**A** = Undetected touch as hand was still on screen: touching the animation/before the next stimulus was presented/before stimulus onset.

**B** = View obscured – parent’s head or hair blocking the view of the infant

**C =** Software did not detect first touch

**D =** Part of the infant’s hand touched a stimulus sensitive area (due to hand position or swiping on screen e.g. little finger catches the other stimulus or hand swiped across stimulus to make response), but clearly aiming for opposite stimuli – only use when this is very clearly the case.

**E =** infant touches the wrong side for balance but is clearly looking at the correct side and touches it on their next response (e.g., immediately after). Code their second response as opposed to their first touch (i.e. the balancing touch) and overwrite software accuracy.

**Reason Terminated Early**

**1** = **Refused to respond to touchscreen** [use this code if they stop touching the screen]

**2** = **Fussiness** [use this code if the infant was still responding but the researcher could see they were tired or upset so stopped anyway]

**3** = **Equipment erro**r [e.g. tablet stopped registering responses or app crashed]

**4** = **Experimenter error** [e.g. no obvious infant/equipment reason, but the experimenter stopped before 20 trials]

**Notes for analysis:**

- For early participants the experimenter did not always cue (i.e. point to or tap) the first trial so it is not always coded as invalid. For consistency with later participants Trial 1 should always be excluded from analysis.

The touch sensitive response area around the buttons is as follows:

**Button size on the iPad: 24 x 13 mm**

**Response area around the buttons (where touches are detected): 44 x 44 mm**


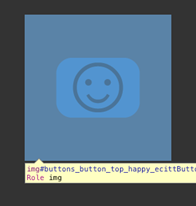

Supplement: Supplementary file 4 [file mmc4.docx]
