# Supplementary material for: Associations between touchscreen exposure and hot and cool inhibitory control in 10-month-old infants
Source: Infant Behav Dev. 2021 Nov;65:101649. doi: 10.1016/j.infbeh.2021.101649 (PMC8641060; doi:10.1016/j.infbeh.2021.101649)
Supplement: Supplementary file 6 [file mmc6.docx]

**Associations between touchscreen exposure and hot and cool inhibitory control in 10-month-old infants**

**Supplementary Materials 6: Assumption and Distribution Checks for Correlation and Regression Analyses**

**Assumption Checks for Correlation Analyses**

The Shapiro-Wilk test was used to assess normality as previous research has shown this test to have a higher power compared to the Kolmogorov-Smirnov test (Razali & Wah, 2011). The Shapiro-Wilk test revealed that only the EEFQ-IC and EEFQ-CEF were normally distributed: (EEFQ-IC: *W*(151) = 1.000, *p* = .920; EEFQ-CEF: *W*(148) = .989, *p =* .325; TUQ: *W*(150) = .924, *p* < .001; EEFQ-Reg: *W*(156) = .916, *p* < .001; ECITT inhibitory score: *W*(128) = .969, *p* = .005, TP: *W*(141) = .489, *p* < .001).

Inspection of the histograms (Supplementary Figures 1.1–1.6) and normal Q-Q plots (Supplementary Figures 2.1–2.6) further support the Shapiro-Wilk test results, where only EEFQ-IC and EEFQ-CEF met the assumption of normality. Therefore, Spearman’s Rank-Order Correlation Coefficient was used to assess the relationship between touchscreen exposure all indices of IC and EF.

**Supplementary Figure 1.1**

*Histogram for the Amount of Touchscreen Exposure Scale (TUQ)*

**
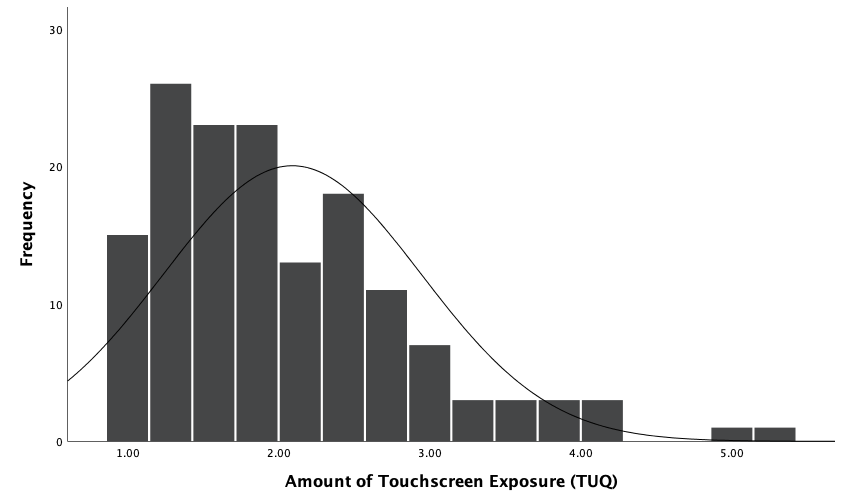
**

**Supplementary Figure 1.2**

*Histogram for Regulation (EEFQ-Reg)*

**
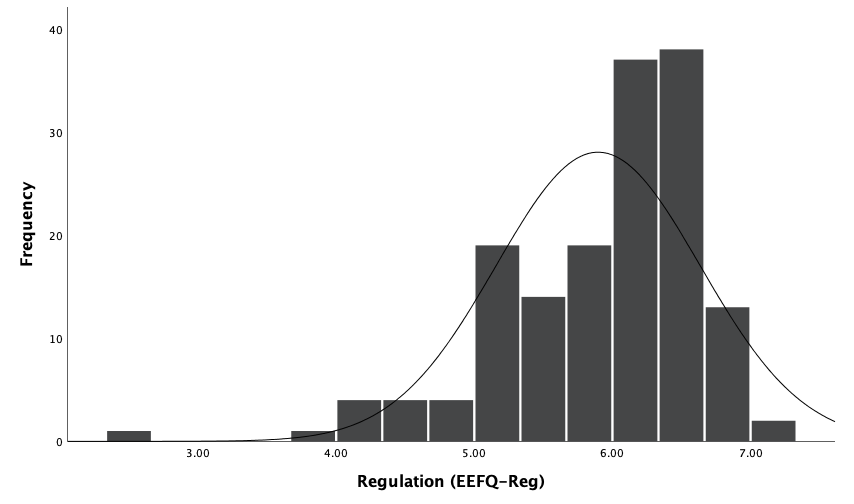
**

**Supplementary Figure 1.3**

*Histogram for Toy Prohibition (TP)*

**
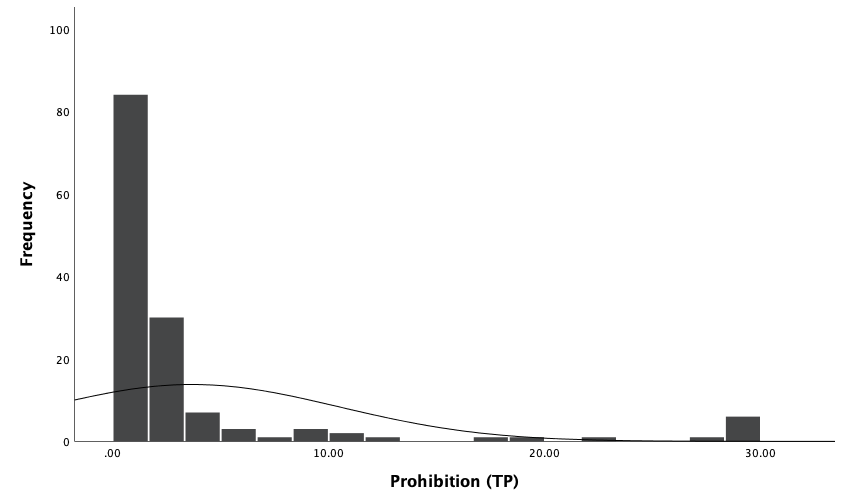
**

**Supplementary Figure 1.4**

*Histogram for Inhibitory Control (EEFQ-IC)*

**
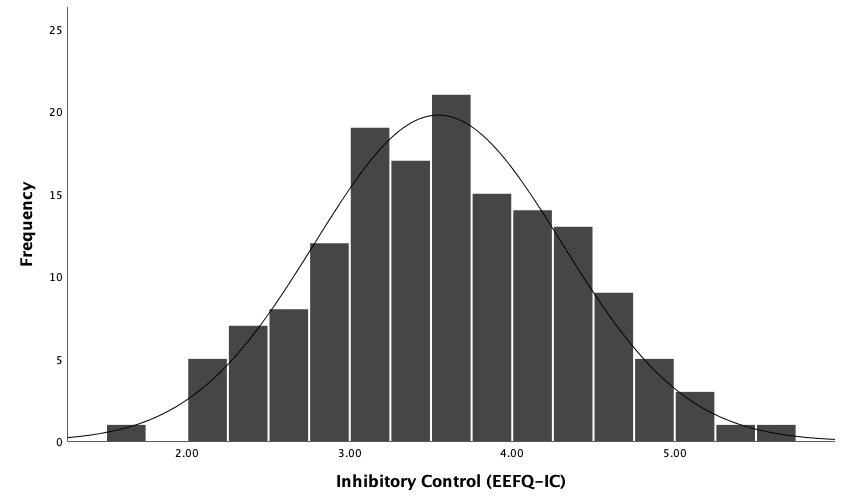
**

**Supplementary Figure 1.5**

*Histogram for Response Inhibition (ECITT inhibitory score)*

**
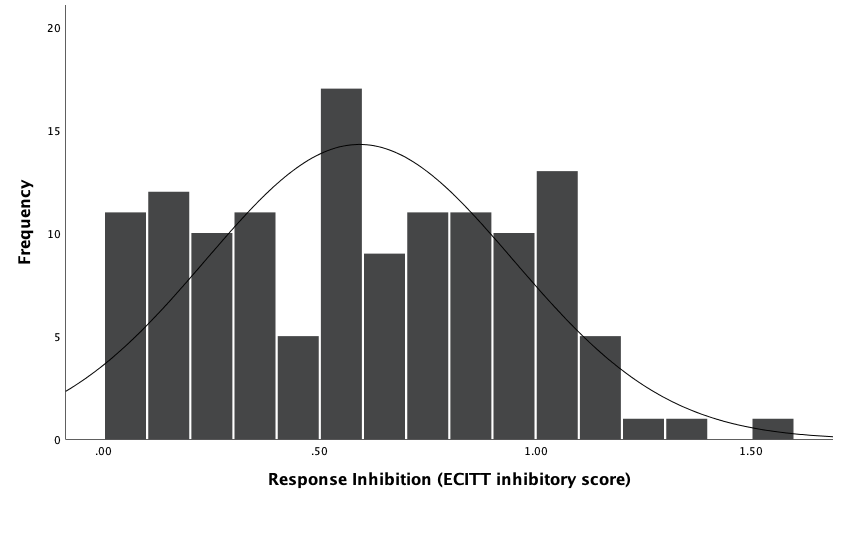
**

**Supplementary Figure 1.6**

*Histogram for Cognitive Executive Function (EEFQ-CEF)*

**
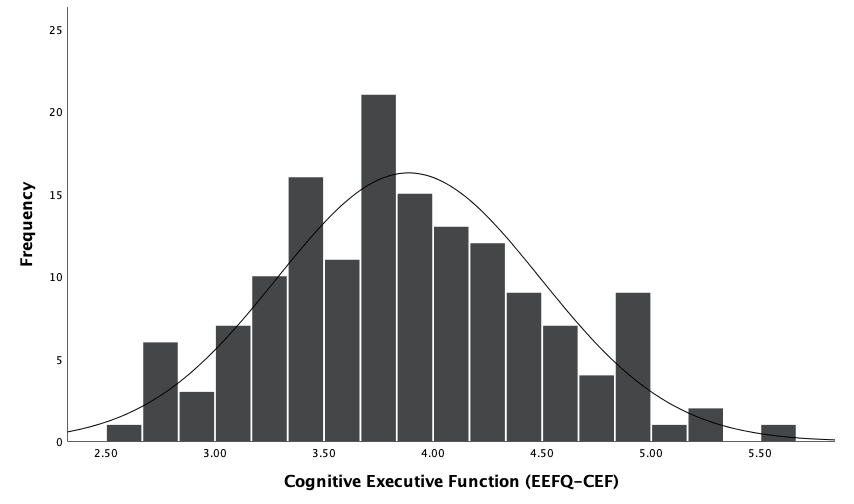
**

**Supplementary Figure 2.1**

*Normal Q-Q Plot for the Amount of Touchscreen Exposure Scale (TUQ)*

**
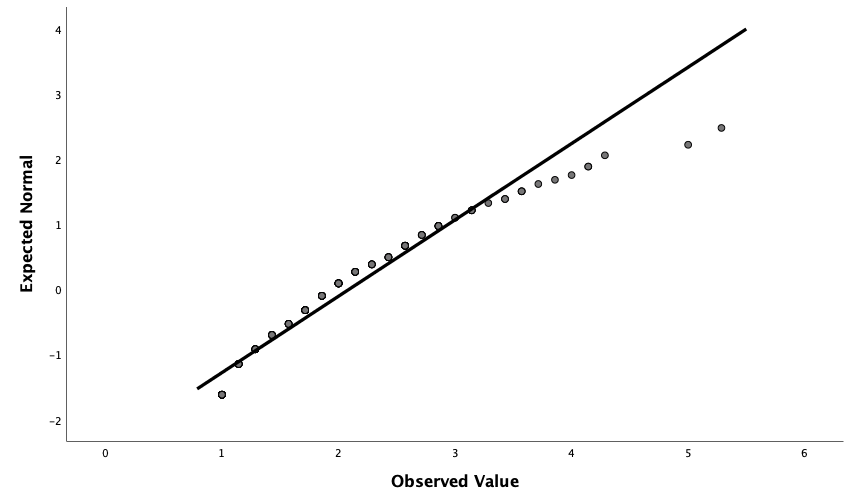
**

**Supplementary Figure 2.2**

*Normal Q-Q Plot for Regulation (EEFQ-Reg)*

**
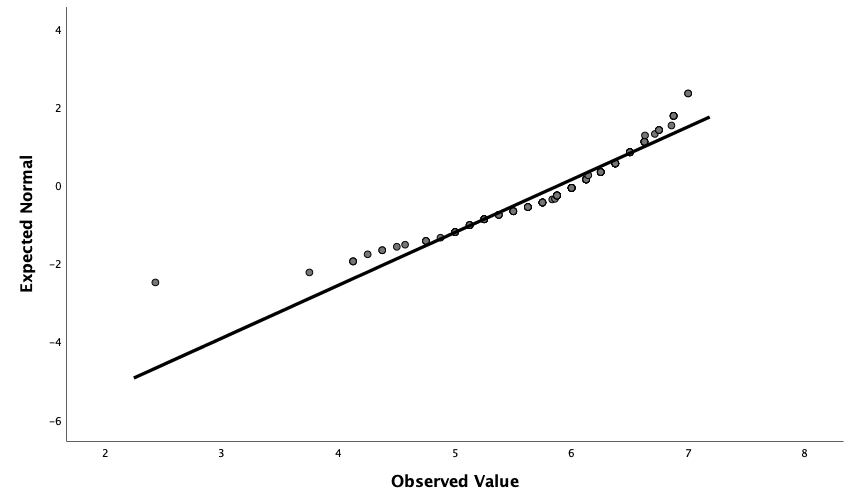
**

**Supplementary Figure 2.3**

*Normal Q-Q Plot for Toy Prohibition (TP)*

**
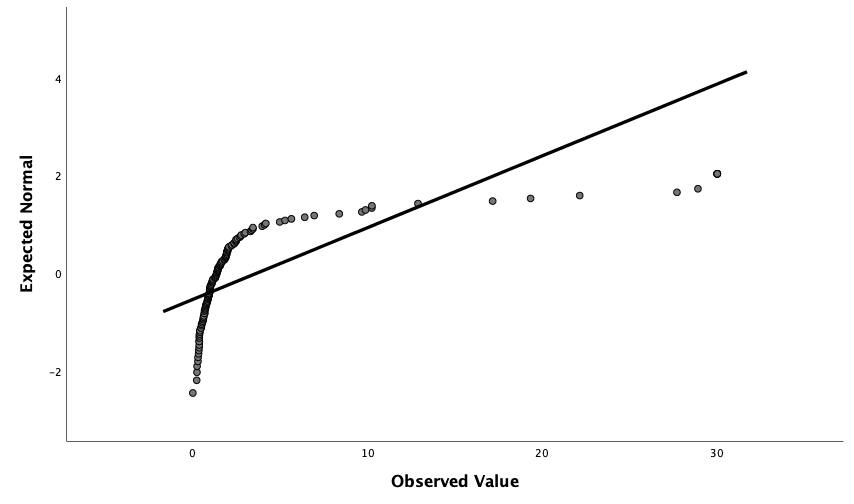
**

**Supplementary Figure 2.4**

*Normal Q-Q Plot for Inhibitory Control (EEFQ-IC)*

**
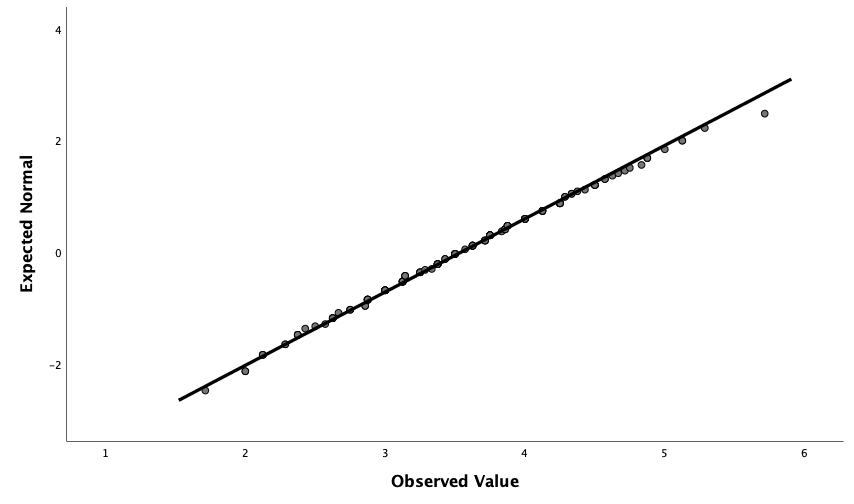
**

**Supplementary Figure 2.5**

*Normal Q-Q Plot for Response Inhibition (ECITT inhibitory score)*

**
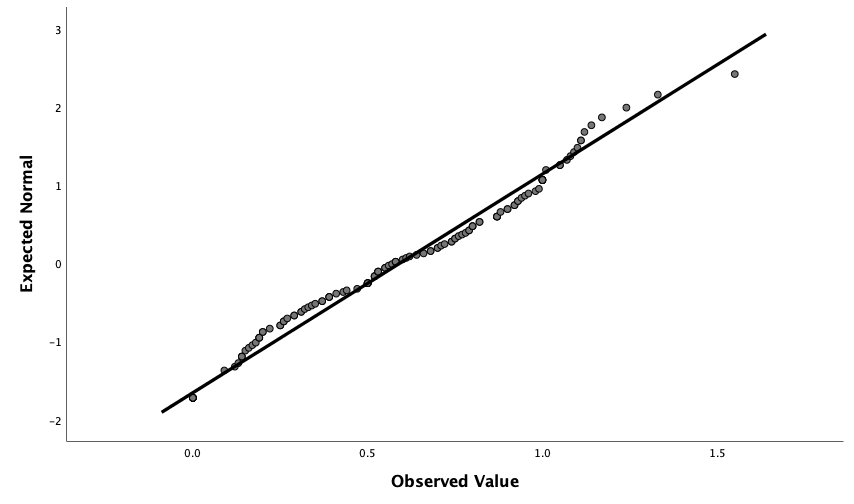
**

**Supplementary Figure 2.6**

*Normal Q-Q Plot for Cognitive Executive Function (EEFQ-CEF)*

**
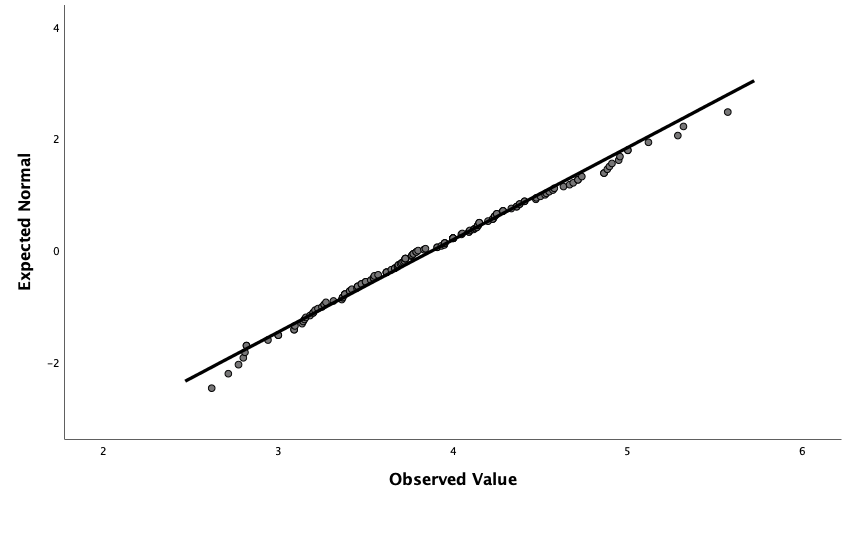
**

**Assumption Checks for Hierarchical Multiple Regression**

***Multicollinearity***

Inspection of correlations using Pearson’s correlation coefficient revealed that none of the independent variables were highly correlated with one another (*r* < .70; Supplementary Table 4). Moreover, the collinearity statistics were all within the expected range, such that the tolerance values were not less than 0.10, and the VIF values were not above 10 (Pallant, 2020; Supplementary Table 5), suggesting that the assumption of multicollinearity was met.

**Supplementary Table 4**

*Bivariate Correlations for All Independent Variables in the Regression Analyses*

|  | 1 | 2 | 3 | 4 | 5 | 6 | 7 |
| --- | --- | --- | --- | --- | --- | --- | --- |
| 1. Amount of Touchscreen Exposure | – |  |  |  |  |  |  |
| 1. Infant’s gender | –.001 | – |  |  |  |  |  |
| 1. Infant’s age | –.064 | –.036 | – |  |  |  |  |
| 1. Mother’s age | –.192^*^ | .034 | .053 | – |  |  |  |
| 1. Father’s age | –.019 | –.008 | .020 | .695^**^ | – |  |  |
| 1. Maternal education | .142 | –.004 | .056 | .179^*^ | .096 | – |  |
| 1. Paternal education | .106 | –.044 | –0.05 | .059 | .013 | .560^**^ | – |

*^**^ p* < .01, *^*^ p* < .05, two-tailed.

**Supplementary Table 5**

*Collinearity Statistics for All Predictors*

|  |  | **Tolerance** | **VIF** |
| --- | --- | --- | --- |
| **Step 1** |  |  |  |
|  | Infant’s gender | 0.992 | 1.008 |
|  | Infant’s age | 0.984 | 1.017 |
|  | Mother’s age | 0.502 | 1.993 |
|  | Father’s age | 0.515 | 1.942 |
|  | Maternal education | 0.659 | 1.518 |
|  | Paternal education | 0.676 | 1.479 |
| **Step 2** |  |  |  |
|  | Infant’s gender | 0.992 | 1.008 |
|  | Infant’s age | 0.980 | 1.020 |
|  | Mother’s age | 0.464 | 2.157 |
|  | Father’s age | 0.499 | 2.002 |
|  | Maternal education | 0.644 | 1.553 |
|  | Paternal education | 0.676 | 1.480 |
|  | Amount of Touchscreen Exposure | 0.899 | 1.112 |

***Normality, Linearity, Homoscedasticity, and Outliers***

Inspection of the normality P-P plots (Supplementary Figure 3.1–3.5) and scatterplots (Supplementary Figure 4.1–4.5) suggest no major deviations from normality with the exception of Prohibition (Supplementary Figure 3.2, 4.2). To determine whether there were any significant outliers, standardized residual values were inspected. Outliers can be detected using Mahalanobis distance values, where any values above a certain critical value (dependent upon the number of independent variables) would be considered a concern. As the current study had seven independent variables, the Mahalanobis distance values should not exceed the critical value of 24.32 (critical value based on Pallant [2020]). None of the cases exceeded this value.

According to Tabachnick and Fidell (2013), cases with standardized residual values above 3.3 or less than –3.3 may also be identified as outliers. For Prohibition, seven outliers were detected (standardized residuals > 3.3). However, as the maximum Cook’s distance value was below 1 (0.157), this suggests that it was not necessary to remove these outliers. For Regulation, one outlier was detected (standardized residual: –4.54). However, as Cook’s distance value was below 1 (0.097), this suggests that this value is unlikely to have significant influence over the results (Tabachnick & Fidell, 2013).

**Supplementary Figure 3.1**

*Normal P-P Plot of Regression Standardized Residuals for Regulation (EEFQ-Reg)*

*
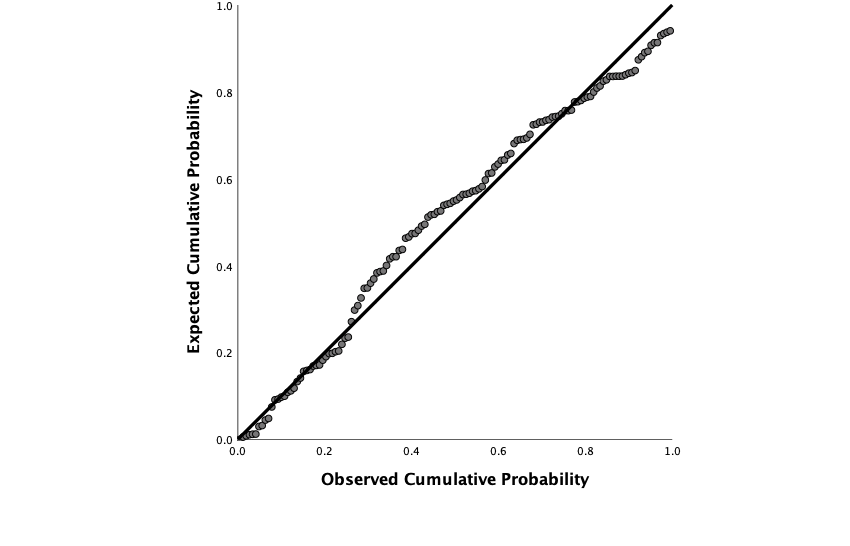
*

**Supplementary Figure 3.2**

*Normal P-P Plot of Regression Standardized Residuals for Toy Prohibition (TP)*

**
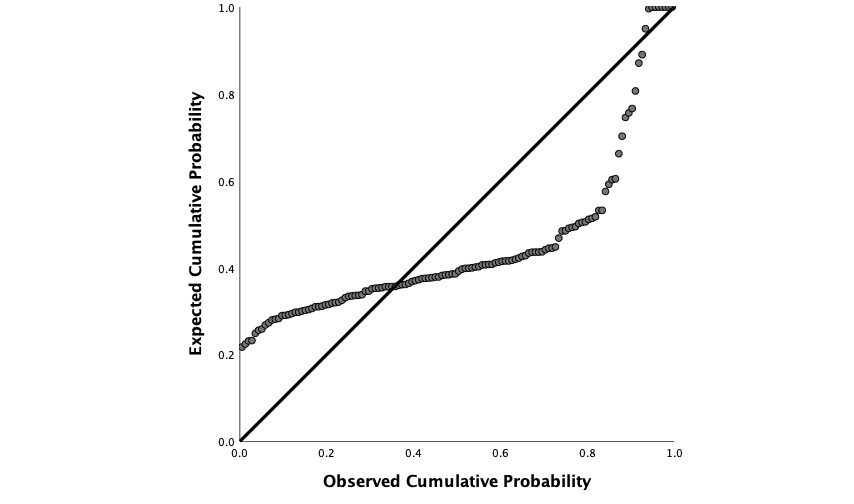
 Supplementary Figure 3.3**

*Normal P-P Plot of Regression Standardized Residuals for Inhibitory Control (EEFQ-IC)*

**
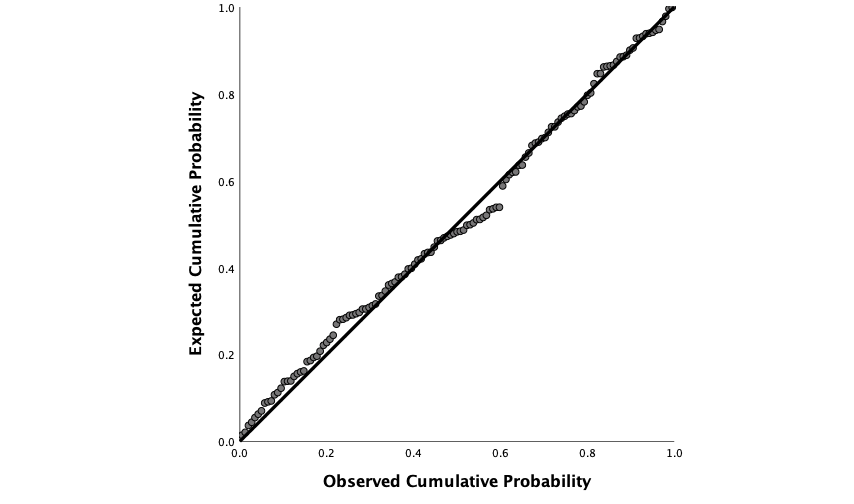
**

**Supplementary Figure 3.4**

*Normal P-P Plot of Regression Standardized Residuals for Response Inhibition (ECITT inhibitory score)*

**
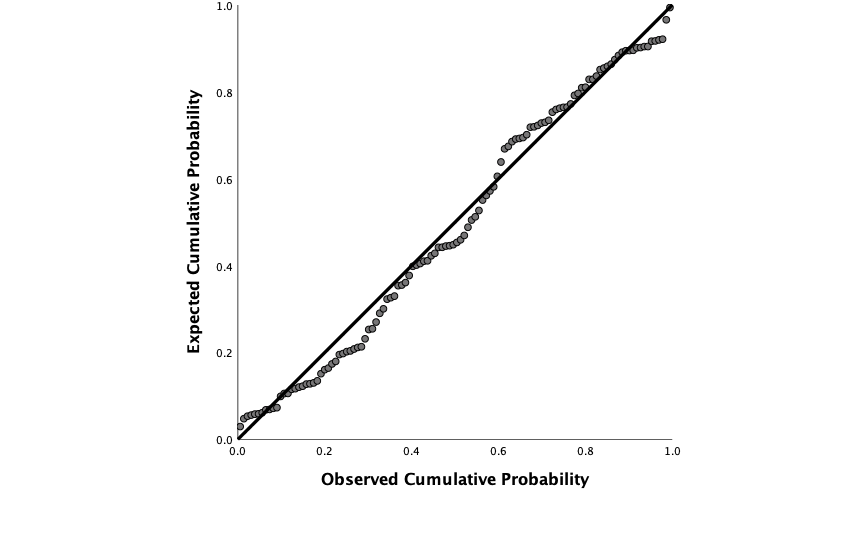
**

**Supplementary Figure 3.5**

*Normal P-P Plot of Regression Standardized Residuals for Cognitive Executive Function (EEFQ-CEF)*


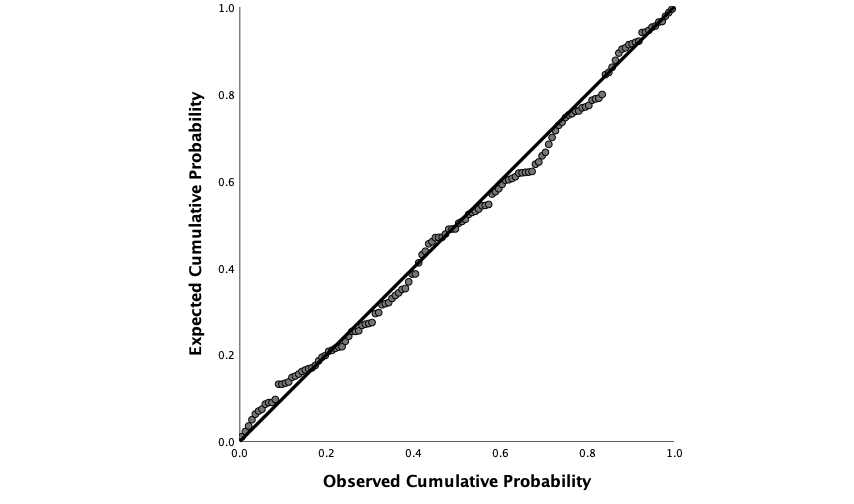


**Supplementary Figure 4.1**

*Scatterplot of Regression Standardized Residuals and Predicted Values for Regulation (EEFQ-Reg)*


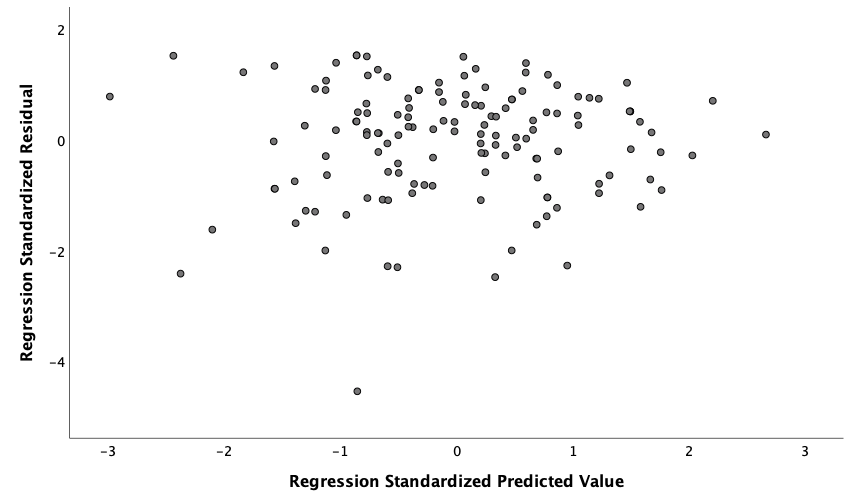


**Supplementary Figure 4.2**

*Scatterplot of Regression Standardized Residuals and Predicted Values for Toy Prohibition (TP)*

*
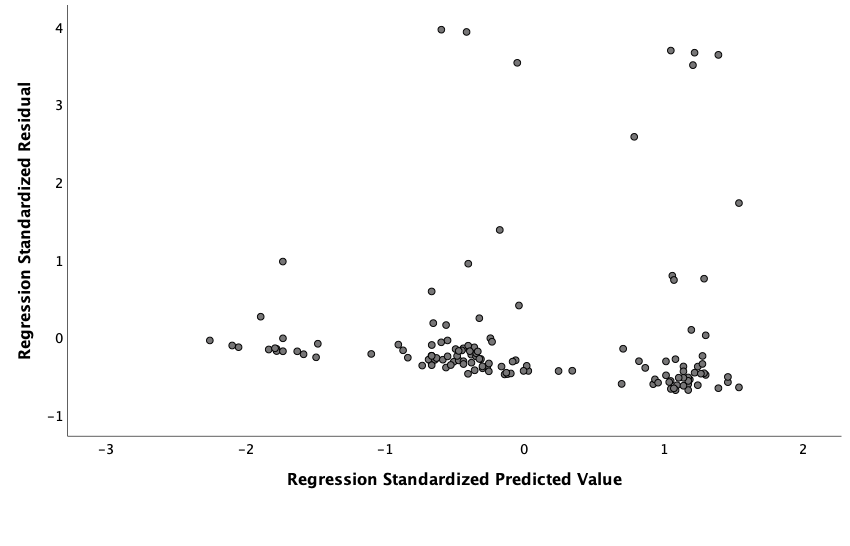
*

**Supplementary Figure 4.3**

*Scatterplot of Regression Standardized Residuals and Predicted Values for Inhibitory Control (EEFQ-IC)*


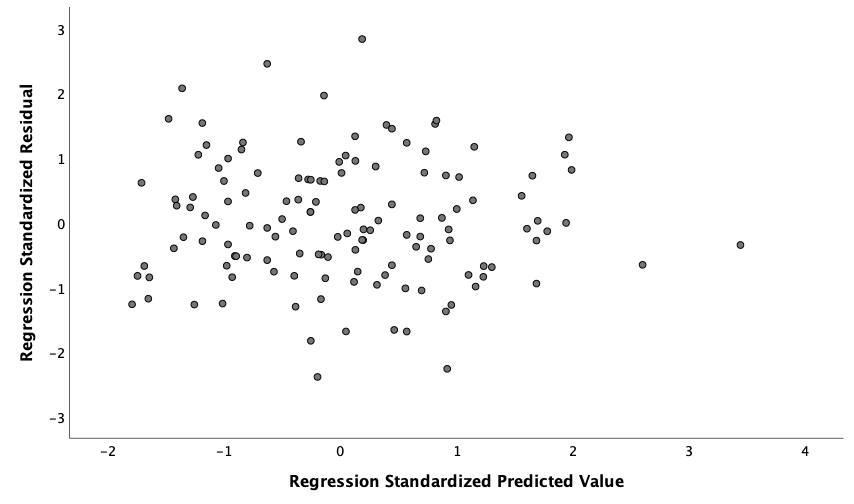


**Supplementary Figure 4.4**

*Scatterplot of Regression Standardized Residuals and Predicted Values for Response Inhibition (ECITT inhibitory score)*

**
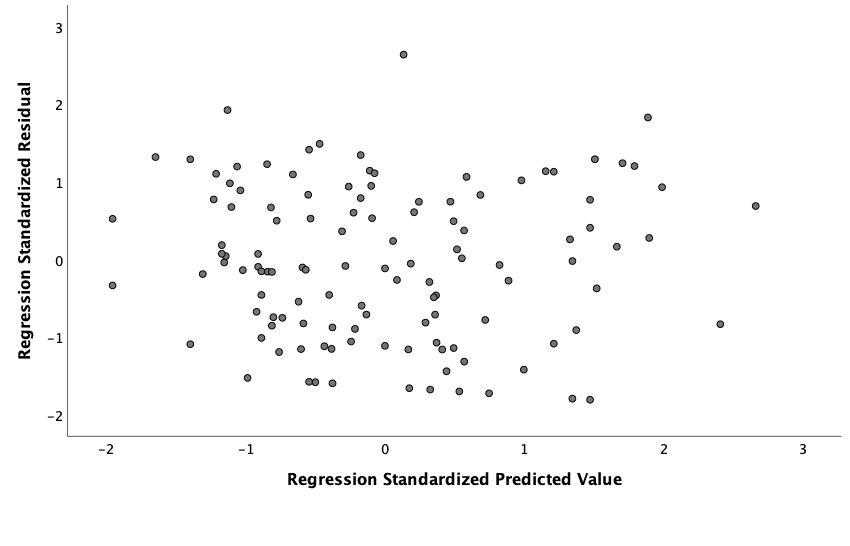
**

**Supplementary Figure 4.5**

*Scatterplot of Regression Standardized Residuals and Predicted Values for Cognitive Executive Function (EEFQ-CEF)*


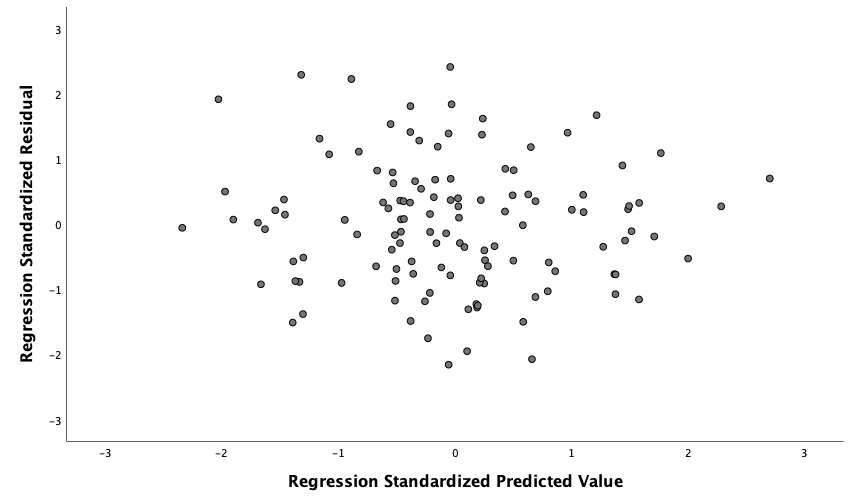


**References**

Pallant, J. (2020). *SPSS survival manual: A step by step guide to data analysis using IBM SPSS* (7th ed.)*.* McGraw-Hill Education.

Razali, N. M., & Wah, Y. B. (2011). Power comparisons of Shapiro-Wilk, Kolmogorov-Smirnov, Lilliefors and Anderson-Darling tests. *Journal of Statistical Modeling and Analytics*, *2*(1), 21–33. <http://www.de.ufpb.br/~ulisses/disciplinas/normality_tests_comparison.pdf>

Tabachnick, B. G., & Fidell, L. S. (2013). *Using multivariate statistics*. (6th ed). Pearson.
