# Supplementary material for: Associations between touchscreen exposure and hot and cool inhibitory control in 10-month-old infants
Source: Infant Behav Dev. 2021 Nov;65:101649. doi: 10.1016/j.infbeh.2021.101649 (PMC8641060; doi:10.1016/j.infbeh.2021.101649)
Supplement: Supplementary file 7 [file mmc7.docx]

**Associations between touchscreen exposure and hot and cool inhibitory control in 10-month-old infants**

**Supplementary Materials 7: Correlation Analyses using ECITT AccD Score**

Additional correlation analyses were performed with alternative indices of the Early Childhood Inhibitory Touchscreen Task (ECITT; Holmboe et al., 2020). Specifically, correlation analyses using the reversed accuracy difference (AccD) score [1 – (prepotent accuracy – inhibitory accuracy)] instead of the inhibitory score were run to examine whether results differed using alternative performance indices for response inhibition. Note that the AccD score was reversed to enable comparability with the inhibitory score and overcome the problem of generating negative correlations resulting from a difference score. Contrary to the inhibitory score used in the primary analyses [(prepotent accuracy – inhibitory accuracy)/prepotent accuracy], this alternative measure of inhibitory performance has not been adjusted and thus does not account for infants' performance on the prepotent trials. Therefore, a *higher* AccD score indicates *better* response inhibition skills.

As shown in Supplementary Table 4, correlations between the Amount of Touchscreen Exposure scale and the accuracy difference (AccD) score were highly consistent with those reported using the inhibitory score as the performance index for response inhibition (for correlations using the inhibitory score, see Table 3). A nominally significant positive association was found between Amount of Exposure and EEFQ-IC (*p* = .031) and EEFQ-CEF (*p* = .036, see Table 3). However, these associations did not survive correction for multiple comparisons using the Benjamini-Hochberg (1995) procedure.

**Supplementary Table 6**

*Correlations Among Key Variables Using ECITT AccD Score*

|  | ***n*** | **1** | **2** | **3** | **4** | **5** | **6** |
| --- | --- | --- | --- | --- | --- | --- | --- |
| 1. Amount of Touchscreen Exposure (TUQ) | 150 | – |  |  |  |  |  |
| 1. Regulation (EEFQ-Reg) | 156 | –.127 | – |  |  |  |  |
| 1. Toy Prohibition (TP) | 141 | .017 | .097 | – |  |  |  |
| 1. Inhibitory Control (EEFQ-IC) | 151 | .182^*^ | –.051 | .149 | – |  |  |
| 1. Response Inhibition (ECITT AccD Score) | 128 | .005 | .098 | .132 | .034 | – |  |
| 1. Cognitive Executive Function (EEFQ-CEF) | 148 | .179^*^ | –.112 | .020 | .749^**^ | –.038 | – |

*Note.* Spearman's Rho correlation coefficients are reported here. TUQ = Touchscreen Use Questionnaire. EEFQ-Reg = Regulation scale. EEFQ-IC = Inhibitory Control scale. ECITT AccD score = Accuracy Difference score. EEFQ-CEF = Cognitive Executive Function score. EEFQ-FX = Flexibility scale. EEFQ-WM = Working Memory scale.
*^**^p* < .01, ^*^*p* < .05, two-tailed, uncorrected for multiple comparisons.

**References**

Benjamini, Y., & Hochberg, Y. (1995). Controlling the false discovery rate: A practical and powerful approach to multiple testing. *Journal of the Royal Statistical Society: Series B (Methodological)*, *57*(1), 289–300. <https://doi.org/10.1111/j.2517-6161.1995.tb02031.x>

Holmboe, K., Larkman, C., de Klerk, C., Simpson, A., Christodoulou, C., & Dvergsdal, H. (2020). *The Early Childhood Inhibitory Touchscreen Task: A new measure of response inhibition in toddlerhood and across the lifespan.* PsyArXiv. <https://doi.org/10.31234/osf.io/k7g4a>
