# Supplementary material for: Associations between touchscreen exposure and hot and cool inhibitory control in 10-month-old infants
Source: Infant Behav Dev. 2021 Nov;65:101649. doi: 10.1016/j.infbeh.2021.101649 (PMC8641060; doi:10.1016/j.infbeh.2021.101649)
Supplement: Supplementary file 8 [file mmc8.docx]

**Associations between touchscreen exposure and hot and cool inhibitory control in 10-month-old infants**

**Supplementary Materials 8: Full Correlation Matrix with Flexibility and Working Memory**

Additional correlation analyses were performed to examine associations between touchscreen exposure and Flexibility and Working Memory, as previous studies have found no effects of screen exposure on Flexibility and Working Memory (McHarg et al., 2020). Detailed descriptions of the Flexibility scale (EEFQ-FX) and Working Memory scale (EEFQ-WM) are presented below (for full details, see Hendry & Holmboe. 2020).

***Flexibility Scale (EEFQ-FX)***

The first item involved parents playing a sorting game with their child, where the child was asked to sort spoons of varying sizes into a small and a large box. However, this item was dropped as 10-month-olds were performing at floor (see Hendry & Holmboe, 2021). The Flexibility scale (EEFQ-FX) reported here therefore includes 7 items, where parents reported the frequency with which their child completed tasks demonstrating cognitive flexibility in the past two weeks (e.g., “try a different way to complete a tricky task without being shown (e.g. when putting shapes in a shape sorter tried different holes)”) on a 7-point scale (1 = *Never*, 7 = *Always*). Scores for this scale were calculated by computing the mean of all items in the scale (applying the 70% minimum threshold). Item scorings were reversed prior to averaging where appropriate. Higher scores thus indicated stronger flexibility. The scale demonstrated somewhat low internal consistency (α = .55). Twenty-nine respondents did not complete the minimum number of items (at least 5 items) to compute the scale mean, and 3 respondents did not complete the EEFQ-FX scale, resulting in a sample of *n* = 130 with EEFQ-FX data available.

***Working Memory Scale (EEFQ-WM)***

The Working Memory (EEFQ-WM) scale includes 8 items reported on a 7-point scale. The first item was a finding game, where parents were instructed to hide a small toy or an object the child enjoyed in one of two opaque containers. This was repeated four times, where the object was hidden in a different container each item. Parents then rated their child’s performance on a 7-point scale (1 = *They didn’t reach to either of the containers at all*, 2 = *They reached to a container once, or more, but didn’t complete the game*, 3 = *They reached to the wrong hiding location each time, 4 = They reached to the correct hiding location once*, 5 = *They reached to the correct hiding location twice*, 6 = *They reached to the correct hiding location three times*, 7 = *They reached to the correct hiding location each time*). For the remaining 7 items, parents reported the frequency with which their child demonstrated abilities requiring working memory in the past two weeks (e.g., “notice (e.g., by expressing surprise or by searching) that something s/he was given a few minutes ago was missing or had changed”) on a 7-point scale (1 = *Never*, 7 = *Always*). Scores for the EEFQ-WM were calculated by computing the mean of all items in the scale (applying the 70% minimum threshold). Item scorings were reversed prior to averaging where appropriate. Higher scores thus indicated stronger working memory. The scale demonstrated adequate, but somewhat low, internal consistency (α = .58). Ten respondents did not complete the minimum number of items (at least 6 items) to compute the scale mean, and 3 respondents did not complete the EEFQ-WM scale, resulting in a sample of *n* = 149 with EEFQ-WM data available.

As shown in Supplementary Table 7, while no significant association was found between touchscreen exposure and EEFQ-WM, a nominally significant positive association was found for EEFQ-FX (*r*_s_ = .178, *p* = .037). However, this did not survive correction using the Benjamini-Hochberg (1995) procedure.

**Supplementary Table 7**

*Full Correlation Matrix with Amount of Exposure, IC, EEFQ-CEF, EEFQ-FX and EEFQ-WM*

|  | ***n*** | **1** | **2** | **3** | **4** | **5** | **6** | **7** | **8** |
| --- | --- | --- | --- | --- | --- | --- | --- | --- | --- |
| 1. Amount of Touchscreen Exposure (TUQ) | 150 | – |  |  |  |  |  |  |  |
| 1. Regulation (EEFQ-Reg) | 156 | –.127 | – |  |  |  |  |  |  |
| 1. Toy Prohibition (TP) | 141 | .017 | .097 | – |  |  |  |  |  |
| 1. Inhibitory Control (EEFQ-IC) | 151 | .182^*^ | –.051 | .149 | – |  |  |  |  |
| 1. Response Inhibition (ECITT inhibitory score) | 128 | –.008 | .084 | .146 | .033 | – |  |  |  |
| 1. Cognitive Executive Function (EEFQ-CEF) | 148 | .179^*^ | –.112 | .020 | .749^**^ | –.049 | – |  |  |
| 1. Flexibility (EEFQ-FX) | 150 | .178^*^ | .008 | –.022 | –.039 | –.095 | .062 | – |  |
| 1. Working Memory (EEFQ-WM) | 160 | –.019 | –.133 | –.079 | .295^**^ | –.167 | .707^**^ | .015 | – |

*Note.* Spearman’s Rho correlation coefficients are reported here. TUQ = Touchscreen Use Questionnaire. EEFQ-Reg = Regulation scale. EEFQ-IC = Inhibitory Control scale. ECITT = Early Childhood Inhibitory Touchscreen Task. EEFQ-CEF = Cognitive Executive Function score. EEFQ-FX = Flexibility scale. EEFQ-WM = Working Memory scale.
*^**^p* < .01, ^*^*p* < .05, two-tailed, uncorrected for multiple comparisons.

**References**

Benjamini, Y., & Hochberg, Y. (1995). Controlling the false discovery rate: A practical and powerful approach to multiple testing. *Journal of the Royal Statistical Society: Series B (Methodological)*, *57*(1), 289–300. <https://doi.org/10.1111/j.2517-6161.1995.tb02031.x>

Hendry, A., & Holmboe, K. (2020). *Development and validation of the Early Executive Functions Questionnaire: A parent-report measure of Executive Function development suitable for 9- to 30-month-olds.* PsyArXiv. <https://doi.org/10.31234/osf.io/rhzkq>

McHarg, G., Ribner, A. D., Devine, R. T., Hughes, C., & NewFAMS Study Team. (2020). Infant screen exposure links to toddlers’ inhibition, but not other EF constructs: A propensity score study. *Infancy*, *25*(2), 205–222. <https://doi.org/10.1111/infa.12325>
