# Supplementary material for: Associations between touchscreen exposure and hot and cool inhibitory control in 10-month-old infants
Source: Infant Behav Dev. 2021 Nov;65:101649. doi: 10.1016/j.infbeh.2021.101649 (PMC8641060; doi:10.1016/j.infbeh.2021.101649)
Supplement: Supplementary file 9 [file mmc9.docx]

**Associations between touchscreen exposure and hot and cool inhibitory control in 10-month-old infants**

**Supplementary Materials 9: Full Hierarchical Multiple Regression Tables**

***Amount of Touchscreen Exposure***

In Supplementary Table 8, we present the full hierarchical multiple regression coefficients for all five of our outcomes and predictors in the final model (Step 2). None of the predictors were significant except for the Amount of Touchscreen Exposure and paternal education with EEFQ-CEF. Paternal education uniquely explained 3.5% of the variance in EEFQ-CEF (*p* = .027). This association was negative, such that higher levels of paternal education predicted lower EEFQ-CEF performance. Due to the exploratory nature of the current study, additional analyses were performed (see below for details).

**Supplementary Table 8**

*Details of Step 2 for Variables Predicting IC and EEFQ-CEF measures*

|  |  | | | |  | | | | |  | **Outcomes** | | | |  |  | | | |  |  | | | |
| --- | --- | --- | --- | --- | --- | --- | --- | --- | --- | --- | --- | --- | --- | --- | --- | --- | --- | --- | --- | --- | --- | --- | --- | --- |
|  | **EEFQ-Reg** | | | | **TP** | | | | |  | **EEFQ-IC** | | | |  | **ECITT** | | | |  | **EEFQ-CEF** | | | |
|  | ***B*** | ***SE B*** | **β** | ***sr^2^*** |  | ***B*** | ***SE B*** | **β** | ***sr^2^*** |  | ***B*** | ***SE B*** | **β** | ***sr^2^*** |  | ***B*** | ***SE B*** | **β** | ***sr^2^*** |  | ***B*** | ***SE B*** | **β** | ***sr^2^*** |
| ***Predictors*** |  |  |  |  |  |  |  |  |  |  |  |  |  |  |  |  |  |  |  |  |  |  |  |  |
| Infant’s gender | –.193 | .129 | –.13 | –.13 |  | 1.849 | 1.228 | .136 | .136 |  | .221 | .130 | .145 | .145 |  | –.031 | .069 | –.043 | –.043 |  | .163 | .102 | .135 | .134 |
| Infant's age | .008 | .010 | .073 | .073 |  | .128 | .093 | .125 | .124 |  | .004 | .010 | .031 | .031 |  | .002 | .005 | .042 | .042 |  | –.001 | .008 | –.01 | –.01 |
| Mother's age | .015 | .021 | .090 | .061 |  | –.053 | .201 | –.035 | –.024 |  | –.035 | .021 | –.205 | –.139 |  | .004 | .011 | .052 | .036 |  | –.023 | .017 | –.17 | –.116 |
| Father’s age | –.001 | .017 | –.008 | –.006 |  | .059 | .160 | .047 | .033 |  | .020 | .017 | .141 | .099 |  | –.002 | .009 | –.032 | –.022 |  | .019 | .013 | .172 | .121 |
| Maternal education | .011 | .025 | .045 | .036 |  | .039 | .241 | .018 | .014 |  | .023 | .026 | .096 | .077 |  | –.018 | .013 | –.157 | –.126 |  | .013 | .020 | .070 | .056 |
| Paternal education | .018 | .024 | .078 | .064 |  | .035 | .229 | .017 | .014 |  | –.045 | .024 | –.194 | –.159 |  | .018 | .013 | .162 | .133 |  | –.042 | .019 | **–.229^*^** | –.188 |
| Amount of Exposure | –.077 | .080 | –.088 | –.084 |  | –.242 | .757 | –.03 | –.029 |  | .124 | .080 | .139 | .132 |  | .025 | .042 | .061 | .057 |  | .136 | .063 | **.192^*^** | .182 |

*Note.* EEFQ-Reg = Regulation scale. TP = Toy Prohibition. EEFQ-IC = Inhibitory Control scale. ECITT = Early Childhood Inhibitory Touchscreen Task. EEFQ-CEF = Cognitive Executive Function. *sr^2^* = squared semi-partial correlation. Step 1 = Infant’s gender, infant’s age, mother’s age, father’s age, maternal education (in years), paternal education (in years). Step 2 = Amount of Touchscreen Exposure.

*^*^p* < .05, highlighted in bold.

***Additional Analyses***

As shown in Supplementary Table 8, an unexpected significant negative association between paternal education and EEFQ-CEF was found in the hierarchical multiple regression model involving Amount of Touchscreen Exposure. For this reason, we conducted an additional regression analysis excluding paternal education. Full details are now presented in Supplementary Table 9. This was performed to determine whether the association between Amount of Touchscreen Exposure and EEFQ-CEF remained when the effect of paternal education on EEFQ-CEF was not included in the model.

Once paternal education was removed, the model as a whole was not significant, *F*(6, 131) = 2.03, *p* = .066. Step 1 sociodemographic variables explained 1.5% of the variance in EEFQ-CEF; this was not significant, *F*(5, 132) = 1.49, *p* = .197. Adding the Amount of Touchscreen Exposure in Step 2 explained an additional 3.2% of variation in EEFQ-CEF*, F*(1, 131) = 4.54, *p* = .035. As the entry of the Amount of Touchscreen Exposure variable in Step 2 explains a similar amount of variance in EEFQ-CEF to when paternal education is included in the model, these findings suggest that the effect of touchscreen exposure on EEFQ-CEF was not entirely driven by paternal education.

**Supplementary Table 9**

*Hierarchical Multiple Regression Tables*

|  | **1** | | | |  | **2** | | | |
| --- | --- | --- | --- | --- | --- | --- | --- | --- | --- |
|  | ***B*** | ***SE B*** | **β** | ***sr^2^*** |  | ***B*** | ***SE B*** | **β** | ***sr^2^*** |
| ***Predictors*** |  |  |  |  |  |  |  |  |  |
| Infant's gender | .178 | .102 | .148 | .147 |  | .175 | .101 | .145 | .145 |
| Infant's age | .000 | .008 | –.003 | –.003 |  | .001 | .008 | .008 | .008 |
| Mother's age | –.032 | .016 | –.240 | –.170 |  | –.023 | .017 | –.168 | –.114 |
| Father's age | .025 | .013 | .223 | .160 |  | .020 | .013 | .179 | .127 |
| Maternal education | –.005 | .016 | –.024 | –.023 |  | –.011 | .017 | –.060 | –.058 |
| Amount of Exposure | – | – | – | – |  | .133 | .062 | **.188^*^** | .178 |

*Note.* Step 1 = Infant’s gender, infant’s age, mother’s age, father’s age, maternal education (in years), paternal education (in years). Step 2 = Amount of Touchscreen Exposure.

*^*^p* < .05, highlighted in bold.

***Age of Initial Touchscreen Exposure***

**Supplementary Table 10.1**

*Details of Step 2 for “Watching/Looking at Videos/Photos” from the Age of Initial Touchscreen Exposure Scale*

|  |  | |  |  | |  | **Outcomes** | |  |  | |  |  | |
| --- | --- | --- | --- | --- | --- | --- | --- | --- | --- | --- | --- | --- | --- | --- |
|  | **EEFQ-Reg** | |  | **TP** | |  | **EEFQ-IC** | |  | **ECITT** | |  | **EEFQ-CEF** | |
|  | **β** | ***sr^2^*** |  | **β** | ***sr^2^*** |  | **β** | ***sr^2^*** |  | **β** | ***sr^2^*** |  | **β** | ***sr^2^*** |
| ***Predictors*** |  |  |  |  |  |  |  |  |  |  |  |  |  |  |
| Infant’s gender | –.147 | –.142 |  | .129 | .125 |  | .159 | .154 |  | –.003 | –.003 |  | .139 | .134 |
| Infant's age | .054 | .052 |  | .140 | .136 |  | .040 | .039 |  | .068 | .066 |  | .001 | .001 |
| Mother's age | .097 | .067 |  | .001 | .000 |  | –.236 | –.162 |  | .085 | .059 |  | –.214 | –.147 |
| Father’s age | –.018 | –.013 |  | .017 | .012 |  | .168 | .117 |  | –.069 | –.048 |  | .201 | .141 |
| Maternal education | .051 | .041 |  | .023 | .019 |  | .123 | .098 |  | –.164 | –.131 |  | .097 | .077 |
| Paternal education | .039 | .031 |  | .012 | .009 |  | –.187 | –.149 |  | .199 | .158 |  | –.213 | –.17 |
| Scroll/swipe | .044 | .040 |  | –.064 | –.059 |  | –.044 | –.041 |  | –.201 | –.186 |  | .010 | .010 |
| Video chat | .080 | .074 |  | .091 | .084 |  | –.006 | –.006 |  | .014 | .013 |  | .012 | .011 |
| Playing games | –.159 | –.139 |  | .140 | .123 |  | –.034 | –.03 |  | .176 | .153 |  | .025 | .022 |
| Drawing/scribbling | –.03 | –.028 |  | .024 | .022 |  | .080 | .074 |  | –.038 | –.035 |  | .018 | .017 |
| Watch/look at videos/photos | –.05 | –.046 |  | –.098 | –.089 |  | .152 | .138 |  | .081 | .074 |  | .103 | .094 |

*Note.* EEFQ-Reg = Regulation scale. TP = Toy Prohibition. EEFQ-IC = Inhibitory Control scale. ECITT = Early Childhood Inhibitory Touchscreen Task. EEFQ-CEF = Cognitive Executive Function. *sr^2^* = squared semi-partial correlation. Step 1 = Infant’s gender, infant’s age, mother’s age, father’s age, maternal education (in years), paternal education (in years), scroll/swipe, video chat, playing games, drawing/scribbling. Step 2 = Watch/look at videos/photos.

**Supplementary Table 10.2**

*Details of Step 2 for “Scroll/swipe” from the Age of Initial Touchscreen Exposure Scale*

|  |  | |  |  | |  | **Outcomes** | |  |  | |  |  | |
| --- | --- | --- | --- | --- | --- | --- | --- | --- | --- | --- | --- | --- | --- | --- |
|  | **EEFQ-Reg** | |  | **TP** | |  | **EEFQ-IC** | |  | **ECITT** | |  | **EEFQ-CEF** | |
|  | **β** | ***sr^2^*** |  | **β** | ***sr^2^*** |  | **β** | ***sr^2^*** |  | **β** | ***sr^2^*** |  | **β** | ***sr^2^*** |
| ***Predictors*** |  |  |  |  |  |  |  |  |  |  |  |  |  |  |
| Infant’s gender | –.147 | –.142 |  | .129 | .125 |  | .159 | .154 |  | –.003 | –.003 |  | .139 | .134 |
| Infant's age | .054 | .052 |  | .140 | .136 |  | .040 | .039 |  | .068 | .066 |  | .001 | .001 |
| Mother's age | .097 | .067 |  | .001 | .000 |  | –.236 | –.162 |  | .085 | .059 |  | –.214 | –.147 |
| Father’s age | –.018 | –.013 |  | .017 | .012 |  | .168 | .117 |  | –.069 | –.048 |  | .201 | .141 |
| Maternal education | .051 | .041 |  | .023 | .019 |  | .123 | .098 |  | –.164 | –.131 |  | .097 | .077 |
| Paternal education | .039 | .031 |  | .012 | .009 |  | –.187 | –.149 |  | .199 | .158 |  | –.213 | –.17 |
| Watch/look at videos/photos | –.05 | –.046 |  | –.098 | –.089 |  | .152 | .138 |  | .081 | .074 |  | .103 | .094 |
| Video chat | .080 | .074 |  | .091 | .084 |  | –.006 | –.006 |  | .014 | .013 |  | .012 | .011 |
| Playing games | –.159 | –.139 |  | .140 | .123 |  | –.034 | –.03 |  | .176 | .153 |  | .025 | .022 |
| Drawing/scribbling | –.03 | –.028 |  | .024 | .022 |  | .080 | .074 |  | –.038 | –.035 |  | .018 | .017 |
| Scroll/swipe | .044 | .040 |  | –.064 | –.059 |  | –.044 | –.041 |  | –.201 | –.186 |  | .010 | .010 |

*Note.* EEFQ-Reg = Regulation scale. TP = Toy Prohibition. EEFQ-IC = Inhibitory Control scale. ECITT = Early Childhood Inhibitory Touchscreen Task. EEFQ-CEF = Cognitive Executive Function. *sr^2^* = squared semi-partial correlations. Step 1 = Infant’s gender, infant’s age, mother’s age, father’s age, maternal education (in years), paternal education (in years), watch/look at videos/photos, video chat, playing games, drawing/scribbling. Step 2 = Scroll/swipe.

**Supplementary Table 10.3**

*Details of Step 2 for “Video chat” from the Age of Initial Touchscreen Exposure Scale*

|  |  | |  |  | |  | **Outcomes** | |  |  | |  |  | |
| --- | --- | --- | --- | --- | --- | --- | --- | --- | --- | --- | --- | --- | --- | --- |
|  | **EEFQ-Reg** | |  | **TP** | |  | **EEFQ-IC** | |  | **ECITT** | |  | **EEFQ-CEF** | |
|  | **β** | ***sr^2^*** |  | **β** | ***sr^2^*** |  | **β** | ***sr^2^*** |  | **β** | ***sr^2^*** |  | **β** | ***sr^2^*** |
| ***Predictors*** |  |  |  |  |  |  |  |  |  |  |  |  |  |  |
| Infant’s gender | –.147 | –.142 |  | .129 | .125 |  | .159 | .154 |  | –.003 | –.003 |  | .139 | .134 |
| Infant's age | .054 | .052 |  | .140 | .136 |  | .040 | .039 |  | .068 | .066 |  | .001 | .001 |
| Mother's age | .097 | .067 |  | .001 | .000 |  | –.236 | –.162 |  | .085 | .059 |  | –.214 | –.147 |
| Father’s age | –.018 | –.013 |  | .017 | .012 |  | .168 | .117 |  | –.069 | –.048 |  | .201 | .141 |
| Maternal education | .051 | .041 |  | .023 | .019 |  | .123 | .098 |  | –.164 | –.131 |  | .097 | .077 |
| Paternal education | .039 | .031 |  | .012 | .009 |  | –.187 | –.149 |  | .199 | .158 |  | –.213 | –.17 |
| Watch/look at videos/photos | .044 | .040 |  | –.064 | –.059 |  | –.044 | –.041 |  | –.201 | –.186 |  | .010 | .010 |
| Scroll/swipe | –.05 | –.046 |  | –.098 | –.089 |  | .152 | .138 |  | .081 | .074 |  | .103 | .094 |
| Playing games | –.159 | –.139 |  | .140 | .123 |  | –.034 | –.03 |  | .176 | .153 |  | .025 | .022 |
| Drawing/scribbling | –.03 | –.028 |  | .024 | .022 |  | .080 | .074 |  | –.038 | –.035 |  | .018 | .017 |
| Video chat | .080 | .074 |  | .091 | .084 |  | –.006 | –.006 |  | .014 | .013 |  | .012 | .011 |

*Note.* EEFQ-Reg = Regulation scale. TP = Toy Prohibition. EEFQ-IC = Inhibitory Control scale. ECITT = Early Childhood Inhibitory Touchscreen Task. EEFQ-CEF = Cognitive Executive Function. *sr^2^* = squared semi-partial correlations. Step 1 = Infant’s gender, infant’s age, mother’s age, father’s age, maternal education (in years), paternal education (in years), watch/look at videos/photos, playing games, drawing/scribbling. Step 2 = Video chat.

**Supplementary Table 10.4**

*Details of Step 2 for “Playing games” from the Age of Initial Touchscreen Exposure Scale*

|  |  | |  |  | |  | **Outcomes** | |  |  | |  |  | |
| --- | --- | --- | --- | --- | --- | --- | --- | --- | --- | --- | --- | --- | --- | --- |
|  | **EEFQ-Reg** | |  | **TP** | |  | **EEFQ-IC** | |  | **ECITT** | |  | **EEFQ-CEF** | |
|  | **β** | ***sr^2^*** |  | **β** | ***sr^2^*** |  | **β** | ***sr^2^*** |  | **β** | ***sr^2^*** |  | **β** | ***sr^2^*** |
| ***Predictors*** |  |  |  |  |  |  |  |  |  |  |  |  |  |  |
| Infant’s gender | –.147 | –.142 |  | .129 | .125 |  | .159 | .154 |  | –.003 | –.003 |  | .139 | .134 |
| Infant's age | .054 | .052 |  | .140 | .136 |  | .040 | .039 |  | .068 | .066 |  | .001 | .001 |
| Mother's age | .097 | .067 |  | .001 | .000 |  | –.236 | –.162 |  | .085 | .059 |  | –.214 | –.147 |
| Father’s age | –.018 | –.013 |  | .017 | .012 |  | .168 | .117 |  | –.069 | –.048 |  | .201 | .141 |
| Maternal education | .051 | .041 |  | .023 | .019 |  | .123 | .098 |  | –.164 | –.131 |  | .097 | .077 |
| Paternal education | .039 | .031 |  | .012 | .009 |  | –.187 | –.149 |  | .199 | .158 |  | –.213 | –.17 |
| Watch/look at videos/photos | .044 | .040 |  | –.064 | –.059 |  | –.044 | –.041 |  | –.201 | –.186 |  | .010 | .010 |
| Scroll/swipe | .080 | .074 |  | .091 | .084 |  | –.006 | –.006 |  | .014 | .013 |  | .012 | .011 |
| Video chat | –.05 | –.046 |  | –.098 | –.089 |  | .152 | .138 |  | .081 | .074 |  | .103 | .094 |
| Drawing/scribbling | –.03 | –.028 |  | .024 | .022 |  | .080 | .074 |  | –.038 | –.035 |  | .018 | .017 |
| Playing games | –.159 | –.139 |  | .140 | .123 |  | –.034 | –.03 |  | .176 | .153 |  | .025 | .022 |

*Note.* EEFQ-Reg = Regulation scale. TP = Toy Prohibition. EEFQ-IC = Inhibitory Control scale. ECITT = Early Childhood Inhibitory Touchscreen Task. EEFQ-CEF = Cognitive Executive Function. *sr^2^* = squared semi-partial correlations. Step 1 = Infant’s gender, infant’s age, mother’s age, father’s age, maternal education (in years), paternal education (in years), watch/look at videos/photos, scroll/swipe, video chat, drawing/scribbling. Step 2 = Playing games

**Supplementary Table 10.5**

*Details of Step 2 for “Drawing/scribbling from the Age of Initial Touchscreen Exposure Scale*

|  |  | |  |  | |  | **Outcomes** | |  |  | |  |  | |
| --- | --- | --- | --- | --- | --- | --- | --- | --- | --- | --- | --- | --- | --- | --- |
|  | **EEFQ-Reg** | |  | **TP** | |  | **EEFQ-IC** | |  | **ECITT** | |  | **EEFQ-CEF** | |
|  | **β** | ***sr^2^*** |  | **β** | ***sr^2^*** |  | **β** | ***sr^2^*** |  | **β** | ***sr^2^*** |  | **β** | ***sr^2^*** |
| ***Predictors*** |  |  |  |  |  |  |  |  |  |  |  |  |  |  |
| Infant’s gender | –.147 | –.142 |  | .129 | .125 |  | .159 | .154 |  | –.003 | –.003 |  | .139 | .134 |
| Infant's age | .054 | .052 |  | .140 | .136 |  | .040 | .039 |  | .068 | .066 |  | .001 | .001 |
| Mother's age | .097 | .067 |  | .001 | .000 |  | –.236 | –.162 |  | .085 | .059 |  | –.214 | –.147 |
| Father’s age | –.018 | –.013 |  | .017 | .012 |  | .168 | .117 |  | –.069 | –.048 |  | .201 | .141 |
| Maternal education | .051 | .041 |  | .023 | .019 |  | .123 | .098 |  | –.164 | –.131 |  | .097 | .077 |
| Paternal education | .039 | .031 |  | .012 | .009 |  | –.187 | –.149 |  | .199 | .158 |  | –.213 | –.17 |
| Watch/look at videos/photos | .044 | .040 |  | –.064 | –.059 |  | –.044 | –.041 |  | –.201 | –.186 |  | .010 | .010 |
| Scroll/swipe | .080 | .074 |  | .091 | .084 |  | –.006 | –.006 |  | .014 | .013 |  | .012 | .011 |
| Video chat | –.159 | –.139 |  | .140 | .123 |  | –.034 | –.03 |  | .176 | .153 |  | .025 | .022 |
| Playing games | –.05 | –.046 |  | –.098 | –.089 |  | .152 | .138 |  | .081 | .074 |  | .103 | .094 |
| Drawing/scribbling | –.03 | –.028 |  | .024 | .022 |  | .080 | .074 |  | –.038 | –.035 |  | .018 | .017 |

*Note.* EEFQ-Reg = Regulation scale. TP = Toy Prohibition. EEFQ-IC = Inhibitory Control scale. ECITT = Early Childhood Inhibitory Touchscreen Task. EEFQ-CEF = Cognitive Executive Function. *sr^2^* = squared semi-partial correlations. Step 1 = Infant’s gender, infant’s age, mother’s age, father’s age, maternal education (in years), paternal education (in years), watch/look at videos/photos, scroll/swipe, video chat, playing games. Step 2 = Drawing/scribbling.
